# The cingulum bundle: Anatomy, function, and dysfunction

**DOI:** 10.1016/j.neubiorev.2018.05.008

**Published:** 2018-09

**Authors:** Emma J. Bubb, Claudia Metzler-Baddeley, John P. Aggleton

**Affiliations:** School of Psychology, Cardiff University, 70 Park Place, Cardiff, CF10 3AT, Wales, UK

**Keywords:** Aging, Alzheimer’s disease, Amygdala, Cingulate gyrus, Diffusion imaging, Emotion, Hippocampus, Memory, Prefrontal cortex, Psychiatry, Retrosplenial cortex, White matter

## Abstract

The cingulum bundle is a prominent white matter tract that interconnects frontal, parietal, and medial temporal sites, while also linking subcortical nuclei to the cingulate gyrus. Despite its apparent continuity, the cingulum’s composition continually changes as fibres join and leave the bundle. To help understand its complex structure, this review begins with detailed, comparative descriptions of the multiple connections comprising the cingulum bundle. Next, the impact of cingulum bundle damage in rats, monkeys, and humans is analysed. Despite causing extensive anatomical disconnections, cingulum bundle lesions typically produce only mild deficits, highlighting the importance of parallel pathways and the distributed nature of its various functions. Meanwhile, non-invasive imaging implicates the cingulum bundle in executive control, emotion, pain (dorsal cingulum), and episodic memory (parahippocampal cingulum), while clinical studies reveal cingulum abnormalities in numerous conditions, including schizophrenia, depression, post-traumatic stress disorder, obsessive compulsive disorder, autism spectrum disorder, Mild Cognitive Impairment, and Alzheimer’s disease. Understanding the seemingly diverse contributions of the cingulum will require better ways of isolating pathways within this highly complex tract.

## Highlights

• Detailed descriptions of connections comprising the cingulum bundle.
• Impact of cingulum bundle damage in rats, monkeys, and humans.
• Imaging evidence of cingulum abnormalities in multiple psychiatric conditions.
• Analyses of changing functions along the length of the cingulum.
• Contrasting effects of fornix and cingulum bundle damage on cognition.

## 1 Introduction

**Fig. 1:**
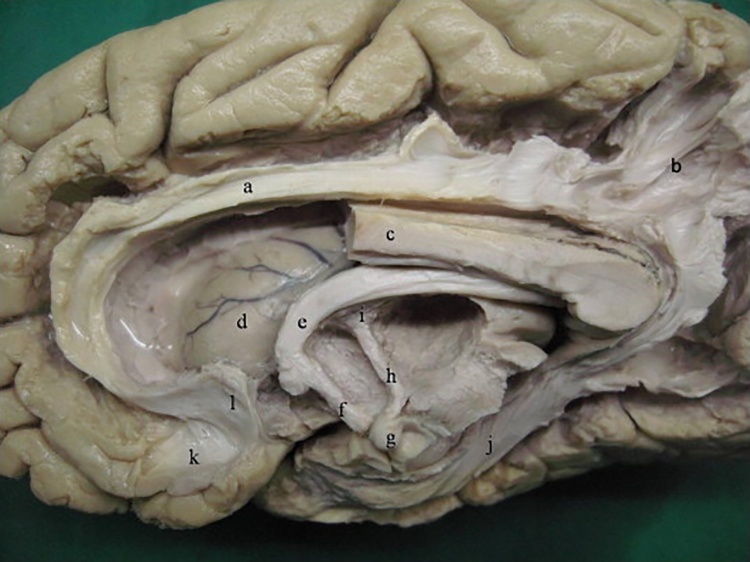
Human brain (medial aspect) after partial dissection (the anterior half of the corpus callosum has been removed), showing major limbic tracts, including parts of the cingulum. Labels: a, cingulum; b, cingulum fibres entering parietal cortex; c, corpus callosum; d, head of caudate nucleus; e, body of the fornix; f, columns of the fornix; g, mammillary body; h, mammillothalamic tract; i, anterior nucleus of the thalamus; j, parahippocampal radiation of the cingulum; k, paraolfactory gyrus; l, paraterminal gyrus. (From Shah et al., 2012, with permission).

The cingulum bundle is one of the most distinctive fibre tracts in the brain, forming a near-complete ring from the orbital frontal cortices, along the dorsal surface of the corpus callosum, then down the temporal lobe towards the pole (Fig. 1). It was Reil (1809) who probably first appreciated the full extent of the tract, though the name ‘cingulum’ is credited to Burdach (1822). While alternative terms have appeared (Swanson, 2014), the name cingulum bundle persists. This may be because ‘cingulum’, the Latin word for an encircling structure such as a girdle or a belt, is so appropriate.

**Fig. 2:**
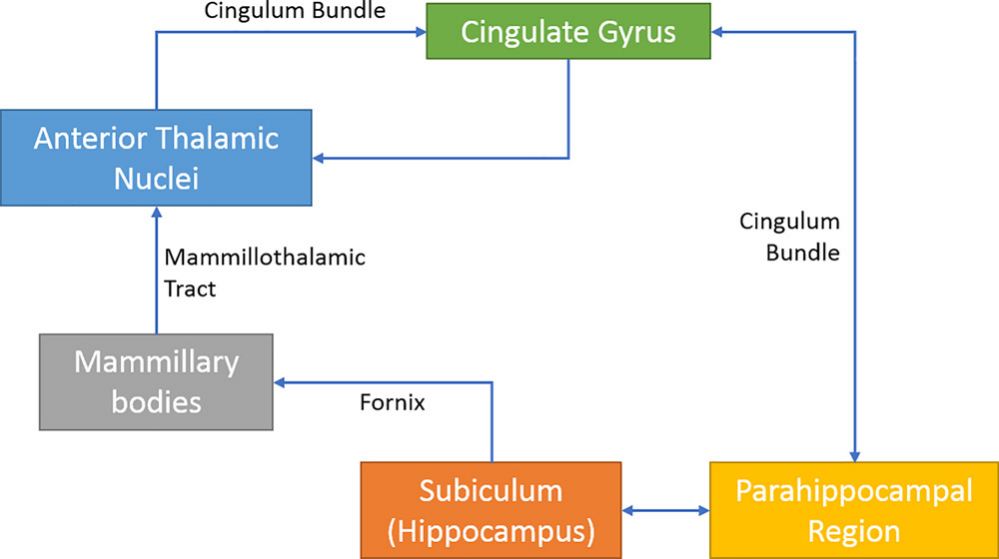
Schematic diagram of Papez circuit (Papez, 1937), showing the central position of the cingulum bundle.

The cingulum’s proximity to the “grand lobe limbique” of Broca (1878) immediately pointed to their close relationship. This cortical relationship was clarified by Beevor (1891) who realised that fibres continuously join and leave the cingulum, emphasising its affinity with the cingulate gyrus. Interest in the cingulum was heightened by Papez (1937), who incorporated the bundle in his influential model of emotion (Fig. 2). Subsequently, the cingulum was seen as a core part of the limbic system (Dalgleish, 2004; MacLean, 1949). One consequence was that the tract became a target for psychosurgical procedures (Section 3.3). More recently, MRI-based evidence of cingulum dysfunctions in a wide range of neurological and psychiatric disorders (Section 4) has boosted further interest in this fibre bundle. Nevertheless, attempts to integrate anatomical and functional knowledge about this tract remain rare, yet this integration is needed to understand this highly complex pathway.

## 2 Structure & connections

### 2.1 Structure

The cingulum is not a unitary pathway. It comprises both short and long sagittal association fibres. In addition, other cingulum fibres radiate across the tract to reach cortical and subcortical sites (Yakolev et al., 1961). Among its sagittal connections are many short cortico-cortical association fibres (‘U-fibres’) that interlink medial parts of the frontal, parietal, and temporal lobes (Schmahmann and Pandya, 2006; Yakolev et al., 1961). Consequently, few, if any, connections extend the entire length of the tract (Heilbronner and Haber, 2014). Interestingly, Cajal (1901; see Schmahmann and Pandya, 2006) thought that the cingulum bundle was predominantly composed of cingulate fibres that head in either a rostral or caudal direction, with the majority bifurcating to go in both directions.

### 2.2 Connections

The following sections concern those connections that comprise the cingulum bundle. The principal findings come from animal experiments, where axonal tracers have helped to visualise projections down to the level of single neurons. The final section provides a comparison across different species.

#### 2.2.1 Rat

**Fig. 3:**
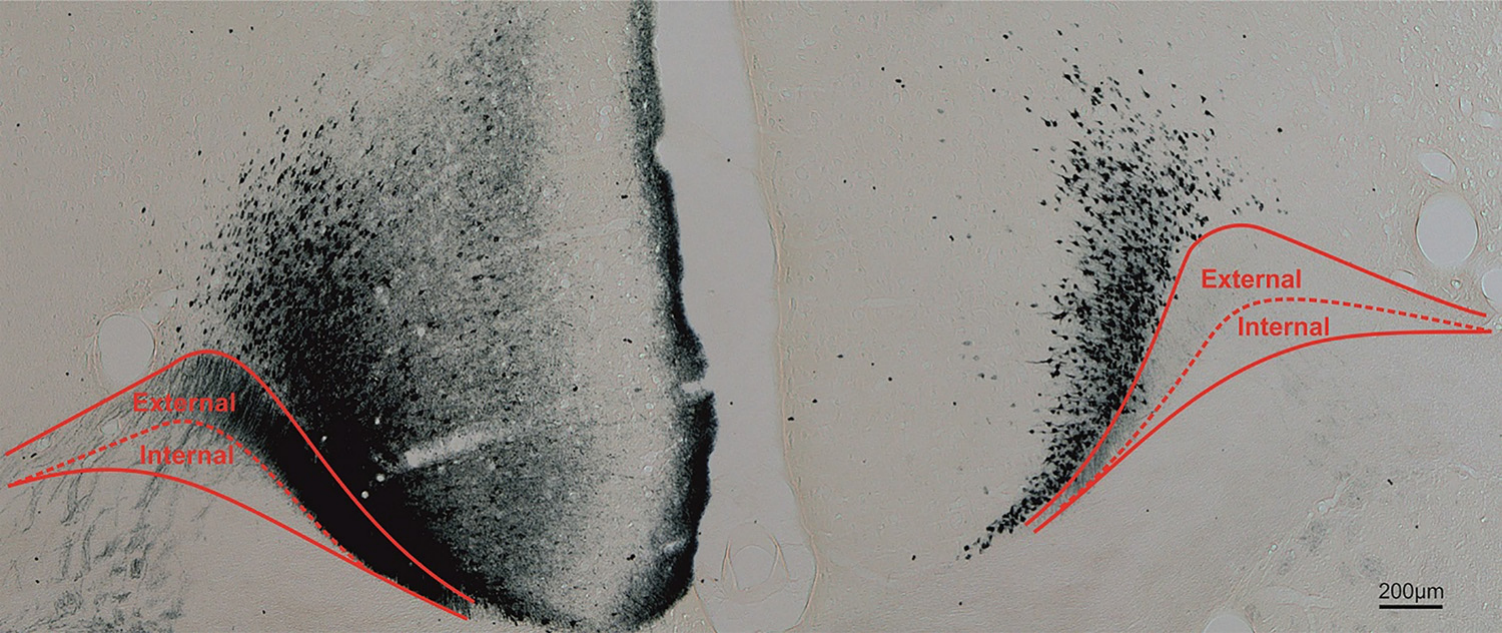
Coronal section from rat brain with anterior thalamic injection of wheat germ agglutin (WGA) in left hemisphere. Labelled anterior thalamic fibres join the external medullary stratum (Domesick, 1970) of the medial cingulum bundle to reach the cingulate cortex. The lack of corresponding fibres in the right hemisphere is because the thalamo-cortical projections remain ipsilateral, although the reciprocal cortico-thalamic projections are bilateral (Mathiasen et al., 2017). For methods, see Amin et al., 2010. Scale bar = 200 μm.

**Fig. 4:**
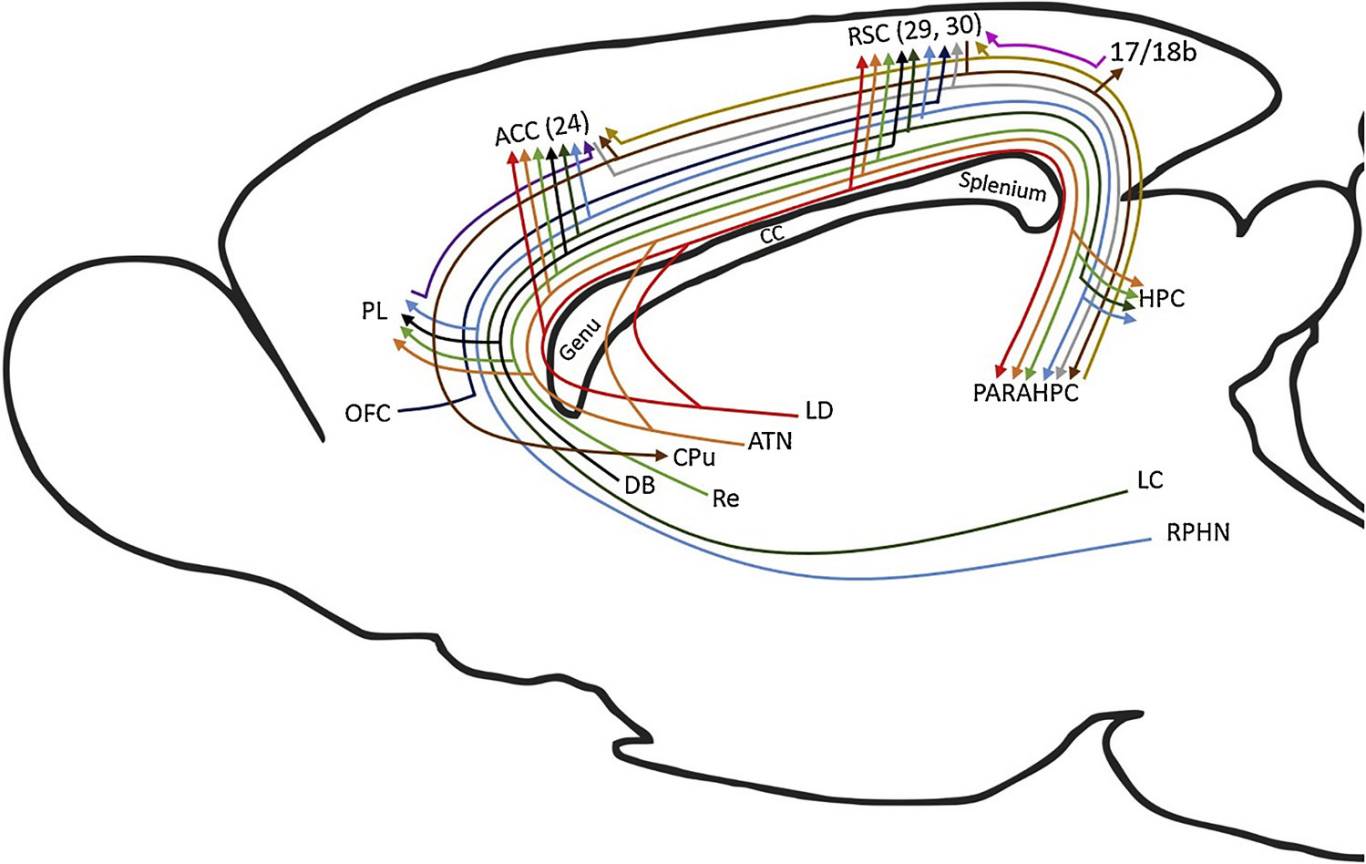
Schematic of rat brain showing connections that provide sagittal fibres to the cingulum bundle. (Note cingulate projections that cross the bundle, e.g., to the anterior thalamic nuclei, are not depicted). The colours help distinguish the multiple pathways. Abbreviations: ACC, anterior cingulate cortex; ATN, anterior thalamic nuclei; CC, corpus callosum; DB, diagonal band; HPC, hippocampus, including subiculum; LC, locus coeruleus; LD laterodorsal thalamic nucleus; OFC, orbital frontal cortex; PARAHPC, parahippocampal region; PL, prelimbic cortex; RPHN, raphe nucleus; RSC, retrosplenial cortex. Note, that dividing lines do not represent collaterals.

Our current knowledge of the rat cingulum bundle originates from studies conducted almost fifty years ago. Using lesion degeneration methods, Domesick (1970) described many anterior thalamic-cingulate projections within the bundle. These projections initially streamed forward from the thalamus to form fascicles in the caudoputamen. Some fibres turned dorsally before reaching the level of the genu to skirt the lateral ventricle, cross through the corpus callosum, and join the external medullary stratum of the cingulum (Domesick, 1970). Other degenerating fibres continued rostrally to the anterior limit of the dorsomedial caudoputamen (some in the internal capsule), then turned medial and dorsal to join the cingulum bundle around the genu of the corpus callosum (Fig. 3, Fig. 4). Together, these efferents formed a basket of thalamo-cingulate fibres, with inputs joining the cingulum at different rostro-caudal levels. Posterior to the splenium, the degenerating thalamic fibres in the cingulum divided to form separate fascicles in caudal retrosplenial and parahippocampal regions (Fig. 4).

The landmark study by Domesick (1970) was, however, limited by how the method would include fibres of passage and the potential problem of distinguishing afferents from efferents. Accordingly, subsequent studies examined thalamic pathways by confining axonal tracers within different nuclei. Fibres from the anterodorsal thalamic nucleus (Van Groen and Wyss, 1995) follow the route described by Domesick (1970) to terminate in granular retrosplenial (area 29), presubicular, and postsubicular cortices, with lighter terminations reaching the entorhinal cortex and subiculum (Van Groen and Wyss, 1990b, c, 1995). Projections from the anteroventral thalamic nucleus follow essentially the same route as anterodorsal efferents, before terminating in the anterior cingulate cortex and area 29, as well as those other areas innervated by the anterodorsal nucleus (Shibata, 1993a, b; Van Groen and Wyss, 1995).

Some fibres from the anteromedial thalamic nucleus join the cingulum before turning rostral to reach medial frontal areas (Shibata, 1993b; Van Groen et al., 1999). Other anteromedial fibres turn caudally in the cingulum to terminate in the posterior half of the anterior cingulate cortex or retrosplenial cortex (both areas 29 and 30), with additional fibres descending behind the splenium to innervate the presubiculum, with lighter terminations reaching entorhinal and perirhinal areas (Shibata, 1993a; Van Groen et al., 1999).

Midline thalamic fibres from the interanteromedial nucleus take a more rostral route, entering the dorsal internal capsule, passing around the rostral limit of the corpus callosum in the cingulum to terminate in frontal and orbital areas (Van Groen et al., 1999). Other interanteromedial fibres turn dorsal and then caudal in the cingulum to reach the anterior cingulate cortex, dysgranular retrosplenial cortex (area 30), the subiculum, and perirhinal cortex (Van Groen et al., 1999). Meanwhile, nucleus reuniens efferents also join the rostral cingulum bundle to innervate prelimbic, anterior cingulate, and retrosplenial cortices (Herkenham, 1978; Wouterlood et al., 1990). Other nucleus reuniens projections continue caudally around the splenium, where they enter the angular bundle and disperse within hippocampal and parahippocampal regions. These fibres innervate the dorsal subiculum, CA1, presubiculum, parasubiculum, the medial entorhinal and perirhinal cortices, although the cingulum is not the only route (Wouterlood et al., 1990). (Note that throughout this review, the subiculum is regarded as part of the hippocampus.)

Fibres from the laterodorsal thalamic nucleus also head rostral and lateral before turning dorsal to enter the cingulum (Fig. 4). Here, most laterodorsal fibres turn caudally to terminate in the retrosplenial cortex (areas 29 and 30), presubiculum, parasubiculum, and postsubiculum, with lighter inputs reaching entorhinal cortex (Van Groen and Wyss, 1990b, c, 1992). A smaller proportion of laterodorsal efferents turn forward in the cingulum to reach anterior cingulate areas (Van Groen and Wyss, 1992). Meanwhile, projections from the mediodorsal thalamic nucleus to the anterior cingulate cortex enter and cross the cingulum around the level of the genu (Leonard, 1969), but do not contribute to the bundle for any length.

Domesick (1969, 1970) also described how the dense, reciprocal corticothalamic projections typically take a different route with respect to the cingulum. Rather than join the sagittal course of the bundle, these connections traverse the cingulum and corpus callosum to reach the internal capsule and then the thalamus. Subsequent studies confirmed this more direct route for retrosplenial, anterior cingulate, and prelimbic projections to the anterior thalamic nuclei (Beckstead, 1979; Shibata, 1998; Shibata and Naito, 2005; Sripanidkulchai and Wyss, 1987; Van Groen and Wyss, 1990a, 1992). There remains, however, the likelihood that a small proportion of cingulate and retrosplenial efferents to the thalamus join the cingulum. Meanwhile, the dense hippocampal and parahippocampal inputs to the anterior thalamic nuclei rely on the fornix and internal capsule (Dillingham et al., 2015; Meibach and Siegel, 1977), rather than the cingulum.

Both the anterior cingulate (area 24) and retrosplenial (areas 29, 30) cortices have dense intrinsic connections, some of which join the cingulum while others take a direct route within the cortex, i.e., dorsal to the tract (Jones et al., 2005; Van Groen and Wyss, 1992, 2003). Likewise, some projections from the anterior cingulate cortex and orbital area to retrosplenial cortex involve the cingulum (Beckstead, 1979; Shibata and Naito, 2008). The cingulum is also the principal route for anterior cingulate and retrosplenial projections (as well as the lighter pregenual cortical projections) to the parahippocampal region, including inputs to the entorhinal cortex, postrhinal cortex, parasubiculum, and presubiculum (Jones and Witter, 2007). In addition, prelimbic projections to the anterior cingulate cortex briefly join the cingulum (Beckstead, 1979).

Retrosplenial cortex has dense interconnections with the adjacent anterior cingulate and secondary motor areas, though it only has weak projections to prelimbic cortex. Some of these same connections join the cingulum (Shibata et al., 2004; Van Groen and Wyss, 1990a, 1992, 2003). Meanwhile, rostral projections from the dysgranular retrosplenial cortex (area 30) to the caudoputamen also join the cingulum (Van Groen and Wyss, 1992). Other cingulum fibres include the reciprocal connections between retrosplenial area 30 and the more visual areas 17 and 18b (Van Groen and Wyss, 1992). Similarly, both areas 29 and 30 have reciprocal connections with the postsubiculum, some joining the cingulum while others take a direct cortico-cortical route (Van Groen and Wyss, 1990c, 2003).

In addition to the thalamus, other subcortical sites contribute to the cingulum. Cholinergic fibres from the diagonal band extend along the cingulum bundle to innervate cingulate and retrosplenial areas, with lighter inputs to frontal areas (Woolf et al., 1986). Noradrenergic fibres from locus coeruleus pass through the anterior thalamus to reach the cingulum, with some fibres terminating in the cingulate cortices and others extending to the hippocampus, including the subiculum (Jones and Moore, 1977; Pasquier and Reinoso-Suarez, 1978; Segal et al., 1973). Additional pontine projections, e.g., from nucleus incertus, course rostrally through the septal region to turn caudally in the cingulum bundle and terminate along the rostrocaudal extent of the cingulate and secondary motor cortices (Goto et al., 2001). Median raphe efferents wrap dorsally around the genu of the corpus callosum (Azmitia and Segal, 1978), joining the cingulum to terminate lightly in frontal cortex, throughout the cingulate cortex, and the entorhinal cortex and dentate gyrus (Pasquier and Reinoso-Suarez, 1978). Finally, some projections reaching the cingulate cortex from the claustrum, lateral hypothalamic area, and amygdala may potentially use the cingulum, though this route does not seem specified (Berk and Finkelstein, 1982; Krettek and Price, 1977; Van Groen and Wyss, 2003).

Fig. 4 depicts those connections that join the cingulum, rather than principally cross the bundle. As a consequence of distinguishing the many cortical sites involved, it might appear from Fig. 4 that the bundle is dominated by cortico-cortical connections. In fact, that part of the bundle above the corpus callosum largely consists of thalamic connections with the cingulate cortices, as well as ascending cholinergic, noradrenergic and serotonergic projections. In contrast, many of the cortico-cortical connections are not only light, but other components link directly across the cortex, i.e., do not join the bundle. A related feature of some cingulum connections is the existence of parallel routes in other pathways, e.g., to parahippocampal areas (Shibata, 1993a; Wouterlood et al., 1990; Zhou and Azmitia, 1983). Overall, the rat cingulum bundle principally provides subcortical connections for cortical regions close to the midline, i.e., with medial frontal, cingulate and parahippocampal cortices, as well as interlinking these same cortical areas.

#### 2.2.2 Nonhuman primate

In their influential analysis, Mufson and Pandya (1984) described three principal groups of cingulum connections. The first group consists of the numerous thalamo-cortical projections that arise from the anterior and laterodorsal thalamic nuclei. As in the rat, many anterior thalamic projections travel rostrally below the caudate nucleus to the anterior limb of the internal capsule, where they turn dorsal to join the cingulum close to the level of the genu. Some thalamic fibres, however, stream directly lateral across the dorsal thalamus, around the lateral ventricle, to enter the cingulum from its lateral side. The anterior thalamic projections joining the cingulum bundle then terminate across the cingulate and retrosplenial cortices (areas 24, 25, 32, 23, 29, 30; Mufson and Pandya, 1984). While a component of the cingulate/retrosplenial inputs from the lateral dorsal thalamic nucleus travels forward to join the cingulum bundle, the majority favour a more direct route around the caudate nucleus and lateral ventricle (Mufson and Pandya, 1984). The return projections from the cingulate cortices to the anterior thalamic nuclei and laterodorsal nucleus take a similar noncingulum route, only crossing the bundle initially (Morris et al., 1999b; Mufson and Mesulam, 1984). Whether a small component of thalamic afferents from the cingulate region travel along the cingulum is difficult to discern, though such fibres are not described (e.g., Baleydier and Mauguiere, 1980; Shibata and Yukie, 2003).

The anteromedial thalamic nucleus is closely connected with the anterior cingulate region (area 24, but also areas 25 and 32). Meanwhile, both the anteroventral and anteromedial nuclei innervate area 23, with the anteroventral nucleus providing most of the inputs to area 30, while both the anterodorsal and anteroventral nuclei innervate area 29 (Bubb et al., 2017; Shibata and Yukie, 2003; Yukie and Shibata, 2009). The laterodorsal nucleus projects to area 23 (Shibata and Yukie, 2003) and 30 (Morris et al., 1999b), with both the laterodorsal and anterior thalamic nuclei also reaching medial parietal area 7 m (precuneus) (Heilbronner and Haber, 2014), while laterodorsal fibres also extend to more lateral parietal areas (Mufson and Pandya, 1984). Other cingulum fibres from the anterior and laterodorsal nuclei reach the parasubiculum, presubiculum, and other parahippocampal cortices (Heilbronner and Haber, 2014; Mufson and Pandya, 1984). There is also a light cingulum projection from the anterior thalamic nuclei to the hippocampal formation (Amaral and Cowan, 1980).

The second principal group comprises cingulate gyrus connections leaving areas 24 and 23 (Mufson and Pandya, 1984). Fibres from area 24 above the corpus callosum join the cingulum and travel forward to terminate in dorsolateral, medial, and orbital prefrontal areas. Other area 24 fibres cross the bundle, some reaching the caudate nucleus and putamen (Baleydier and Mauguiere, 1980), other fibres join the anterior limb of the internal capsule, before entering the thalamus and brainstem. Additional cingulate fibres from area 24 pass along and through the lateral portion of the cingulum bundle to reach the extreme capsule and terminate in the amygdala, perirhinal cortex, insula and superior temporal cortex (Mufson and Pandya, 1984). Rostral area 24 also appears to project to more posterior area 24, as well as area 23, via the cingulum bundle, though there are few inputs to other parietal areas (Pandya et al., 1981; Vogt and Pandya, 1987).

Projections from area 23 that join the cingulum include fibres to lateral parietal sites such as area 7a, the dorsal prelunate, and lateral intraparietal area, with additional fibres reaching the superior temporal sulcus (Kobayashi and Amaral, 2007; Mufson and Pandya, 1984). Other cingulum fibres from area 23 pass caudal to the splenium, to split and re-join, and then enter parahippocampal areas TH and TF, as well as the presubiculum. In return, parahippocampal areas, including the entorhinal and perirhinal cortices, project to area 23 (Baleydier and Mauguiere, 1980; Vogt and Pandya, 1987), presumably via the cingulum. In addition, restricted rostral projections from area 23 reach dorsal prefrontal cortex (areas 9, 46) via the cingulum, but largely avoid adjacent area 24 (Kobayashi and Amaral, 2007; Mufson and Pandya, 1984). Finally, area 23 efferents cross the cingulum bundle and to reach the caudate nucleus (Mufson and Pandya, 1984). Related fibres continue ventrally in the internal capsule to reach thalamic (principally the anterior thalamic nuclei, the lateral dorsal nucleus, and the mediodorsal nucleus) and brainstem targets. These brainstem projections, which arise from along the cingulate gyrus and initially cross the cingulum, terminate topographically in the pontine grey matter (Vilensky and Van Hoesen, 1981).

Although not described by Mufson and Pandya (1984), their second fibre group would have included efferents from retrosplenial cortex (areas 29, 30), many of which join the cingulum. Rostrally directed retrosplenial efferents in the cingulum reach anterior area 23, caudal area 24, as well as areas 46 and adjacent 9 in the dorsolateral prefrontal cortex, with light inputs to frontal areas 6, 8, 10, 11, and 12 (Kobayashi and Amaral 2007; Morris et al., 1999a,b). Some caudally directed retrosplenial efferents that join the cingulum terminate in area 19, while more retrosplenial fibres join the parahippocampal cingulum to reach areas TH and TF, as well as the presubiculum, parasubiculum, and parts of entorhinal cortex (Kobayashi and Amaral, 2007; Morris et al., 1999b). These same areas project back to retrosplenial cortex (areas 29, 30) and area 23, with the inputs from parahippocampal TH and TF involving the cingulum (Bubb et al., 2017). While retrosplenial cortex receives dense, direct inputs from the subiculum, these projections cut directly across the presubiculum, some from the cingulum bundle, others from the alveus (Aggleton et al., 2012). Inputs from the lateral intraparietal area to area 30 (Kobayashi and Amaral, 2003) potentially involve the cingulum.

The third principal group (Mufson and Pandya, 1984) consists of projections from both anterior frontal and posterior parietal regions. Dorsolateral prefrontal cortical areas, including 9, 10, and 46, project via the cingulum bundle to posterior cingulate areas 23, 31, and the retrosplenial cortex, as well as to medial parietal area 7 m and the presubiculum (Goldman-Rakic et al., 1984; Morris et al., 1999a; Selemon and Goldman-Rakic, 1988). In addition, the frontal pole (area 10) reaches targets along the cingulate and retrosplenial cortices via the cingulum (Heilbronner and Haber, 2014). Meanwhile, efferents from parietal area 7 m join the dorsalmost cingulum to terminate in areas 23 and 24 of the cingulate gyrus (Mufson and Pandya, 1984), with a few fibres continuing to dorsal frontal areas 6 and 8 (Petrides and Pandya, 1984). Finally, projections from the caudal inferior parietal lobule join the parahippocampal cingulum to reach the presubiculum (Seltzer and Pandya, 1984).

A later analysis (Heilbronner and Haber, 2014) described how almost all prefrontal cortical areas contribute at least some fibres to the cingulum bundle. Furthermore, some dorsomedial frontal and caudal orbitofrontal areas have fibres within almost all components of the bundle, although the parahippocampal cingulum contains the least frontal fibres. Meanwhile, projections from the posterior cingulate region (including areas 29 and 30) are also found almost throughout the cingulum (Heilbronner and Haber, 2014).

Heilbronner and Haber (2014) also described basolateral amygdala projections that join the subgenual, rostral dorsal, and parahippocampal subdivisions of the cingulum, though not its caudal dorsal subdivision (see Aggleton et al., 2012; Amaral and Price 1984). In particular, efferents from the amygdala to the anterior cingulate cortex join the subgenual and rostral dorsal cingulum, while some amygdala projections to frontal areas 6, 8, and 9 may also involve parts of the bundle (Heilbronner and Haber, 2014). Finally, direct amygdala inputs to the subiculum and prosubiculum (Aggleton, 1986) can involve the parahippocampal cingulum.

**Fig. 5:**
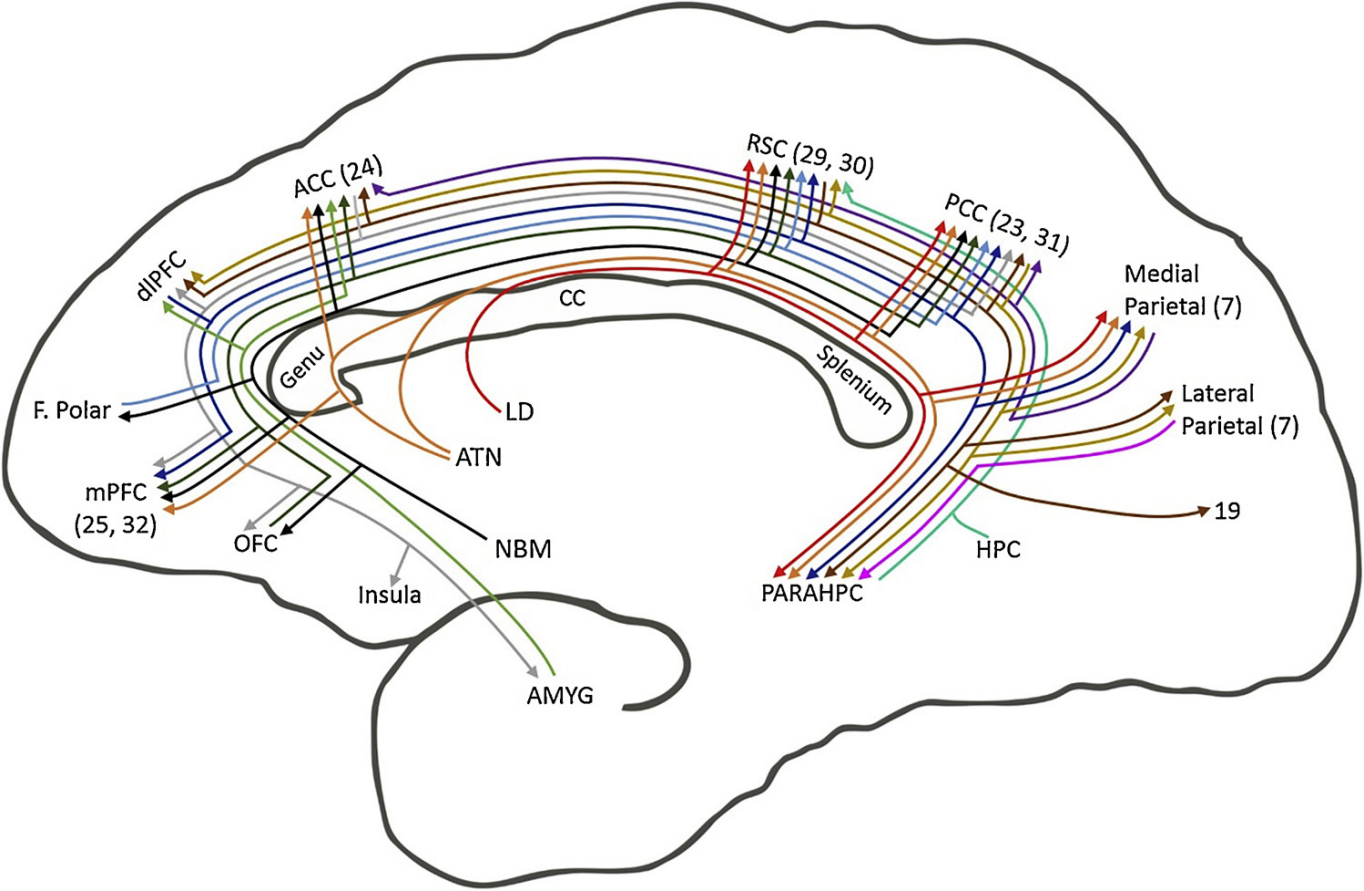
Schematic of macaque monkey brain showing connections that provide sagittal fibres to the cingulum bundle. (Note cingulate projections that cross the bundle, e.g., to the anterior thalamic nuclei, are not depicted). The colours help distinguish the multiple pathways. While it is most likely that additional subcortical projections join the cingulum (see Fig. 4), explicit descriptions are often lacking. Abbreviations: ACC, anterior cingulate cortex; ATN, anterior thalamic nuclei; CC, corpus callosum; HPC, hippocampus, including subiculum; LC, locus coeruleus; LD laterodorsal thalamic nucleus; NBM, nucleus basalis of Meynert; PARAHPC, parahippocampal region; PL, prelimbic cortex; RSC, retrosplenial cortex. Note, that offshoots of lines do not represent actual collaterals.

Many cholinergic fibres from nucleus basalis of Meynert in the basal forebrain join the cingulum to run above the corpus callosum and innervate the length of the cingulate gyrus, as well as superior frontal cortices (Kitt et al., 1987; see also Selden et al., 1998 for corresponding human data). Meanwhile, projections from the mediodorsal thalamic nucleus to areas 24 and 23 potentially join the cingulum (Baleydier and Mauguiere, 1980; Shibata and Yukie, 2003; Vogt et al., 1987). Likewise, other thalamic nuclei with cingulate gyrus interconnections that may involve the cingulum bundle include lateralis posterior, reuniens, parataenialis, centralis densocellularis, centralis latocellularis, paracentralis, parafascularis, limitans, ventralis anterior, and the pulvinar (Baleydier and Mauguiere, 1980; Shibata and Yukie, 2003, 2009; Vogt et al., 1987). Again, projections from locus coeruleus, the raphe nucleus, and other brainstem sites, including the central grey, to the cingulate cortices (Porrino and Goldman-Rakic, 1982) presumably involve the cingulum, though the precise routes of many of the above connections remain to be specified and so are not incorporated in summary Fig. 5.

Like the rat, the monkey cingulum contains many short projection fibres but, in comparison, it innervates more cortical areas. Again, as in the rat, the cingulum connections include noradrenergic, serotonergic, and cholinergic fibres. One noticeable gap in Fig. 5 is between the anterior cingulate and retrosplenial cortices. This gap reflects the presence of a distinct midcingulate cortical area, which is most evident in primate brains (Vogt, 2009) but is also present in rodents (Vogt and Paxinos, 2014). Unfortunately, many of the relevant tracing studies occurred before this area was distinguished, leaving its connections poorly understood and, hence, not illustrated. It is, however, clear from Fig. 5 that no single site dominates the tract, as its component connections shift along the length of the bundle. Consequently, interventions at different levels would be expected to have different outcomes.

#### 2.2.3 Human

Initial anatomical findings came from microdissections and from reconstructions based on cellular and white matter stains. In recent years, major advances have come from non-invasive diffusion weighted magnetic resonance imaging (dMRI), which exploits the Brownian motion of water protons in brain tissue for the in vivo reconstruction, visualisation, and quantification of white matter microstructure. Diffusion tensor imaging (DTI) (Basser et al., 1994; Basser and Pierpaoli, 1996) has been the most influential dMRI method. Given the current and future impact of these methodologies, we will consider them in further detail.

The DTI method fits a tensor with three eigenvectors to the diffusion data. From the tensor a number of indices of white matter microstructural properties can be derived (Pierpaoli and Basser, 1996). For instance, the “mean square displacement” of water molecules can be estimated by averaging the three diffusion tensor eigenvalues, giving the mean diffusivity index (MD). Axial diffusivity (AD) reflects diffusivity along the principal orientation of a fibre tract whilst radial diffusivity (RD) refers to diffusivity perpendicular to the principal fibre orientation. Fractional anisotropy (FA), a metric of the degree of fibre coherence or directionality can be understood as the relative ratio between AD and RD, approaching 0 in isotropic and 1 in anisotropic conditions (Acosta-Cabronero and Nestor, 2014). When interpreting DTI indices as measures of white matter microstructure, it is important to recognise that they are not only influenced by biological properties of white matter such as axon myelin, diameter, and density but also by the orientational complexity and organisation of the fibre architecture (Beaulieu and Allen, 1994). For this reason, it is difficult to interpret differences/changes in FA and diffusivity metrics in terms of any specific biophysical property of white matter (Wheeler-Kingshott and Cercignani, 2009; Jones, 2010). Having said this, DTI metrics are known to be sensitive indices of white matter microstructural changes and the in vivo investigation of the cingulum bundle in humans is predominately based on DTI techniques.

In addition, dMRI allows the reconstruction of white matter bundles by means of fibre tractography (Basser et al., 2000; Tournier et al., 2004; Jeurissen et al., 2011, 2017) or, alternatively, the reconstruction of a white matter skeleton from voxels that exceed a predefined FA threshold with tract based spatial statistics (TBSS) (Smith et al., 2006). Such DTI metric maps can also extract average values for region of interest (ROI) analysis or for whole brain analysis voxel-based analysis (VBA). While DTI based tractography (Basser et al., 2000) has been widely applied, it has difficulties in resolving fibre tracking in areas with mixed fibre orientations due to, for instance, intra-voxel fibre crossing (Jones and Cercignani, 2010; Jeurissen et al., 2017).

Recent research efforts have, therefore, focused on improving fibre tracking techniques by means of i. optimising dMRI data acquisition, for example with high angular resolution diffusion imaging (HARDI) (Tuch et al., 2002) and ii. fibre tracking algorithms, for instance, with spherical deconvolution based techniques that allow the resolution of peaks in fibre orientation functions (Tournier et al., 2004; Tournier et al., 2007; Dell’acqua et al., 2010). Whilst these methods can improve fibre tracking (Jeurissen et al., 2017), it is worth remembering that current shortcomings of dMRI tractography also include an inability to determine the direction of white matter (afferent or efferent), a limitation that is highly problematic for studies of the cingulum bundle. Meanwhile, the problem of disentangling complex fibre architecture (Fischer et al., 2012; Jeurissen et al., 2017) helps to explain why the reconstructed cingulum sometimes appears to extend rostrally beyond the limit of the tract (Heilbronner and Haber, 2014), suggesting that streamlines have jumped into adjacent pathways. These factors show how the choice of dMRI data acquisition, processing, and tracking algorithm methods can influence the status of the derived cingulum bundle.

**Fig. 6:**
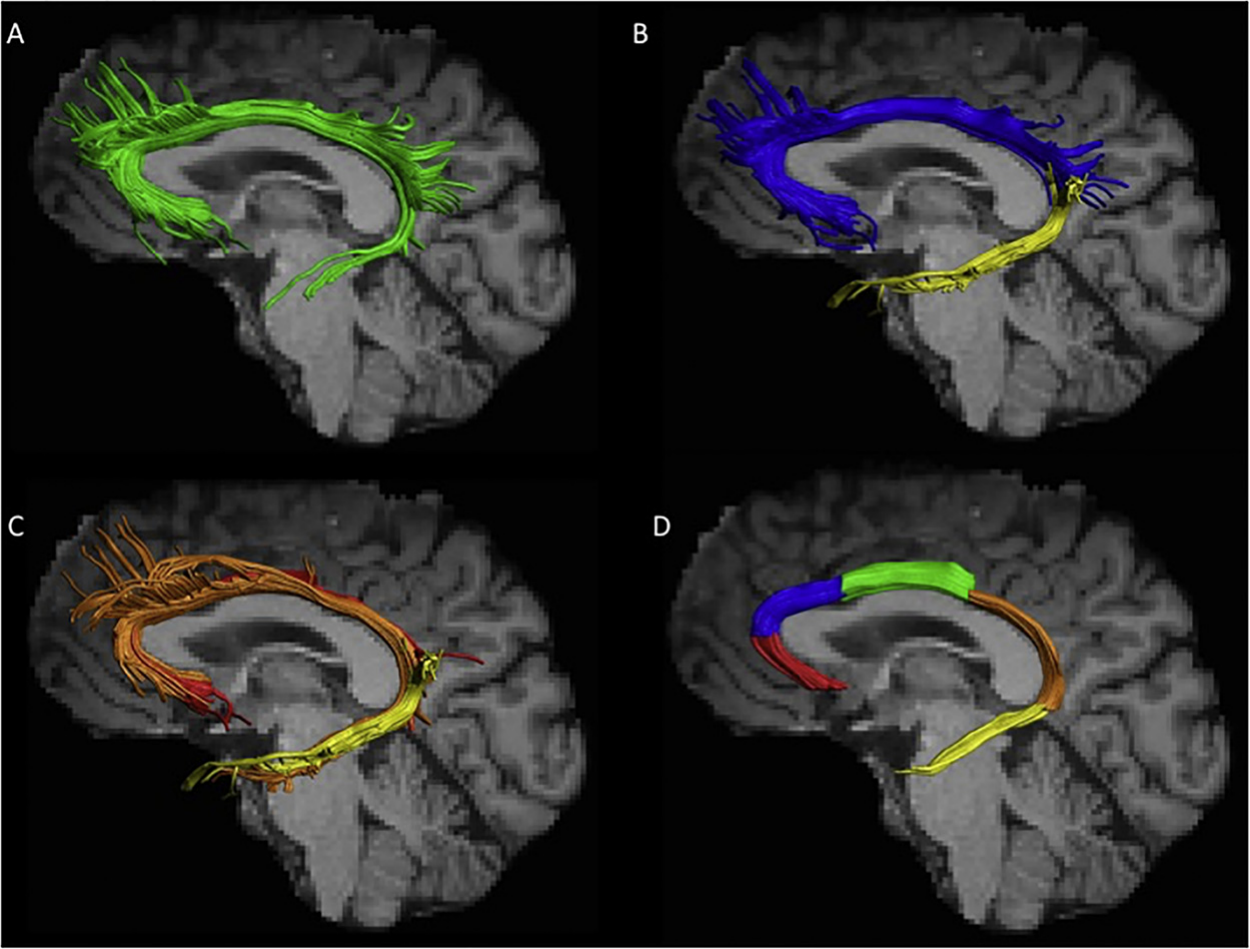
Cingulum bundle reconstructions based on diffusion MRI tractography. Images show the left cingulum for one healthy individual displayed on a T_1_-weighted image. A) The cingulum reconstructed as a single, continuous bundle (green), B) Dorsal (blue) and ventral (yellow) cingulum subdivisions (e.g., Budisavljevic et al., 2015). C) Subgenual (red), retrosplenial (orange), and parahippocampal (yellow) cingulum subdivisions (Jones et al., 2013). D) Proposed subdivisions along the longitudinal axis, with subgenual (red), anterior cingulate (blue), midcingulate (green), retrosplenial (orange), and parahippocampal (yellow) portions. Note that additional streamlines, e.g., in frontal and parietal areas, have been removed (For interpretation of the references to colour in this figure legend, the reader is referred to the web version of this article).

Our literature review reveals much heterogeneity in cingulum reconstructions across different studies. Initial DTI tractography studies portrayed the cingulum as a unitary bundle (e.g., Catani et al., 2002; Xie et al., 2005; see Fig. 6A). However, to better reflect its changing anatomical properties, reconstructions increasingly distinguished between the ‘dorsal’ and ‘ventral’ cingulum, i.e., above or below the splenium (e.g., Budisavljevic et al., 2015; Fig. 6B) which roughly corresponds to the distinction between cingulum cingulate gyrus and cingulum angular bundle (e.g., Ezzati et al., 2016). Further distinctions include the anterior (close to the genu) and posterior (close to the splenium) cingulum. Other researchers using the constrained spherical deconvolution algorithm (Tournier et al., 2004) for fibre tracking have divided the tract three-ways, e.g., into its ‘subgenual’, ‘retrosplenial’ (supracallosal), and ‘parahippocampal’ (ventral) components (Jones et al., 2013; see Fig. 6C). These three subdivisions were found to exhibit distinct FA measures and occupied different medial-lateral positions within the bundle, even in areas where they overlapped (Jones et al., 2013). These differences, which suggest qualitative changes along the length of the tract, add support to similar tract subdivisions used in other dMRI studies (e.g., Concha et al., 2005; Kates et al., 2015; Lin et al., 2014; Metzler-Baddeley et al., 2017).

Given the length and complexity of the cingulum bundle (Fig. 5, Fig. 6) it is to be expected that finer detailed tract subdivisions might provide even better correspondence with potential functional changes. Accordingly, Heilbronner and Haber (2014) used monkey connectivity to help subdivide the primate cingulum into four divisions; subgenual, rostral dorsal (anterior cingulate), caudal dorsal (retrosplenial), and temporal (parahippocampal). Furthermore, some dMRI studies have added additional cingulum subdivisions (Kennis et al., 2016; Metzler-Baddeley et al., 2012b; Whitford et al., 2014). Thus, there is clearly need for a consensus on how best to subdivide the cingulum in dMRI research. Based on current anatomical evidence, we would recommend taking the four subdivisions proposed by Heilbronner and Haber (2014), but adding the midcingulate cortical area (Vogt, 2009) to make five subdivisions (Fig. 6D).

#### 2.2.4 Cross-species comparisons

Although many of the details of the human cingulum bundle remain to be specified, it is possible to make comparisons across the three highlighted species. There seems every reason to believe that the set of subcortical – cortical connections, which appear to dominate the rat cingulum bundle, are duplicated in the primate brain, including humans. Given its importance for the cingulum bundle, it is important to appreciate that there are strong homologies between the cytoarchitecture of the rodent and primate (including human) cingulate cortex, including its major subdivisions (Vogt and Paxinos, 2014).

At the same time, there is an obvious increase in cortico-cortical connectivity within the primate cingulum bundle. Rather than just involving cingulate, medial frontal and parahippocampal areas, there is a marked extension as the tract contains some fibres from across almost all parts of the prefrontal cortex (Heilbronner and Haber, 2014) as well as reaching more dorsal and lateral parts of parietal cortex. These parietal connections include the precuneus and may also include more dorsal parts of the superior parietal lobule (areas PE and PM) and the inferior parietal lobule (PG) (Catani and Thiebaut de Schotten, 2012; Schmahmann and Pandya, 2006). Other lateral parts of the parietal lobe have efferents that cross through the cingulum bundle to reach the cingulate cortices (Schmahmann and Pandya, 2006). In addition to an apparent increase in parietal – frontal connectivity within the bundle, DTI reconstructions often suggest a corresponding increase in parietal – temporal connections within the human parahippocampal cingulum bundle. There is a need to examine further these connections as they may reflect a clear difference with other primates, such as macaques.

The core set of connections found across species has helped to place the cingulum bundle within the limbic system. Unsurprisingly, attempts to understand the functions of its connections have often focussed on emotion and memory. Meanwhile, the greater emphasis on frontal connections in the primate cingulum bundle has led researchers to consider its potential contributions to cognitive control, attention, pain, motor mechanisms, and reward signalling (Beckmann et al., 2009).

## 3 Functional analyses of the cingulum bundle – lesion analyses

### 3.1 Rodent studies

Studies of lesions targeted specifically at the cingulum bundle have predominantly examined pain perception or spatial processing. The former follows from the introduction of anterior cingulotomies for intractable pain in humans (Section 3.3), the second from the many hippocampal and parahippocampal connections within the tract. A limitation of all of these studies is that they are not accompanied by experiments that identify the extent of disconnection caused by the various interventions.

Initial research showed that blockade of the rat cingulum bundle can cause analgesia (Vaccarino and Melzack, 1989; 1992), an effect not due to adjacent cortical inactivation (Vaccarino and Melzack, 1989). Related studies described how cingulum bundle anaesthesia delays the onset of self-mutilation following peripheral neurectomy (Vaccarino and Melzack, 1991; see also Magnusson and Vaccarino, 1996). In contrast, stimulation of the cingulum can precipitate self-mutilation (Pellicer et al., 1999). The finding that electrical stimulation of the cingulum bundle reduces formalin-test pain (Fuchs et al., 1996) was interpreted as a disruption of patterned activity that would normally signal pain.

The contribution of the cingulum bundle to pain perception has been interpreted in different ways. Melzack (2005) regarded the cingulum as part of a widely distributed ‘neuromatrix’, which together provides pain perception. Instead, Vogt (2005) argued for a ‘dual pain system’, which assumes more specific contributions. While part of this effect involves an impact on emotion, it is supposed that there are particular anterior cingulate contributions that reflect pain perception (Vogt, 2005; but see Shackman et al., 2011). This nociceptive information may come from midline and intralaminar thalamic nuclei (Vogt, 2005), helping to explain the significance of the cingulum.

The second topic, spatial memory and navigation, arises from the close links between the cingulum bundle and brain sites known to contribute to spatial processes. These links are detailed in Table 1, which compares the behavioural effects of cingulum bundle lesions with damage to associated areas (anterior cingulate cortex, retrosplenial cortex, anterior thalamic nuclei). The fornix is included in Table 1 as it contains the connections from the hippocampus to the anterior thalamic nuclei. All of the spatial tasks in Table 1 are highly sensitive to hippocampal lesions. The terms ‘reference’ and ‘working’ refer, respectively, to when a fixed piece of information is learnt, or when the information changes across trials/sessions. All cortical lesions were made by cytotoxins, to avoid cingulum bundle damage.

**Table 1:** Comparison of cingulum bundle (CB) lesion effects in rats with other, related brain sites. Symbols: * results from same study as cingulum bundle lesion; √, no lesion effect; X, mild/borderline effect; XX, clear deficit; XXX, severe deficit. Numbers in parenthesis show the number of cingulum lesions in each hemisphere. In two studies (Neave et al., 1996, 1997) there were two groups with cingulum bundle lesions, which differed in the number of lesion placements per hemisphere (one, CB1 or two, CB2). In both studies, the lesions were placed asymmetrically to avoid bilateral cortical damage.

Task	Cingulum Bundle study	Cingulum Bundle	Retrosplenial. Cortex	Anterior Thalamic Nuclei	Fornix	Anterior Cingulate
Water-maze reference acquisition	Warburton et al. 1998 (2)	X	X^1^ X^6^	XXX^2^	XX* XX^2^ XXX^12^	X*
	Harker and Whishaw 2004 (1)	X	X*			
Water-maze working	Harker and Whishaw 2004 (1)	X	X* X^1^	XXX^15^	XXX^14^	
T-maze alternation acquisition	Aggleton et al. 1995 (3)	XXX	√* (ant +post cingulate) X^8^ √^10^	XXX^3^ XXX^7^	XXX* XXX^7^	√^10^ X^13^ marginal
	Neave et al. 1996 (2,1)	X CB2 X CB1				
	Neave et al. 1997 (2,1)	XX CB2 √ CB1			XXX*	
	Warburton et al. 1998 (2)	X			XXX*	
T-maze alternation delays	Aggleton et al. 1995 (3)	XX	√* (ant +post cingulate) √^10^	XX^3^ XXX^4^	XXX^3,4^	√^10^
	Neave et al. 1996 (2,1)	√ CB2 √ CB1 (X when groups combined)				
Cross-maze alternation	Neave et al. 1997 (2,1)	√ CB2 √ CB1		XXX^4^	XXX*	
Delayed nonmatch to position in operant box	Aggleton et al. 1995 (3)	√	√* (ant +post cingulate)	XX^5^	XX*	√^10^
	Neave et al. 1996 (2,1)	√ CB2 √ CB1				
Lever discrimination and reversals	Aggleton et al. 1995 (3)	√	√* (ant +post cingulate)		XX*	√^10^
Radial arm maze (working)	Neave et al. 1997 (2,1)	XX CB2 √ CB1	XX^6^	XXX^9^	XXX*	√^11^
Object recognition	Ennaceur et al. 1997 (3)	√	√* √^6^	√^2^	√*	√*
Object location recognition	Ennaceur et al. 1997 (3)	√	XX* (ant +post cingulate)		XX*	

The superscript numbers refer to appropriate comparison studies: 1. later reanalysis in Vann et al., 2003: 2. Warburton and Aggleton, 1999: 3. Aggleton et al., 1995: 4. Warburton et al., 1997: 5. Aggleton et al., 1991: 6. Vann and Aggleton, 2002: 7. Warburton et al., 1999: 8. Nelson et al., 2015: 9. Aggleton et al., 1996: 10. Neave et al., 1994: 11. Ragozzino et al., 1998: 12. Sutherland and Rodriguez, 1989: 13. Sánchez-Santed et al., 1997: 14. Cassel et al., 1998: 15. Perry et al., 2018.

Several conclusions emerge from Table 1. The first is that cingulum bundle lesions most consistently affect spatial tasks involving allocentric cues, i.e., when the relationships between distal cues specify location. Nevertheless, despite the dense contributions to the bundle from the anterior thalamic projections to the cingulate cortices, cingulum bundle lesions are far less disruptive than anterior thalamic lesions. This difference reveals the relative importance of those anterior thalamic connections that avoid the cingulum bundle, e.g., its inputs from the hippocampal region, the mammillary bodies, and frontal cortices, while also signifying how these thalamic nuclei are a key point of convergence for spatial processing (Bubb et al., 2017).

Table 1 also highlights the close correspondence between the effects of retrosplenial cortex lesions and cingulum bundle lesions on spatial memory. These cingulum effects become more robust as more lesions are placed along the tract. This pattern presumably reflects how cortical fibres join and leave the tract along its length. Consequently, there are additive effects as more anterior cingulate and retrosplenial cortex disconnections occur (see Vann et al., 2003). The related question of whether retrosplenial cortex disconnection largely accounts for the effects of cingulum bundle lesions on spatial tasks was specifically tested by Harker and Whishaw (2002), who compared aspiration retrosplenial lesions that either spared or compromised the bundle. While retrosplenial lesions impaired both reference and working memory tasks in the water maze, these deficits were not exacerbated by additional cingulum bundle damage. This result also matches the finding of similar effects on water-maze tasks after separate retrosplenial and cingulum bundle lesions (Harker and Whishaw, 2004; see Table 1), the tract severance begin made at a rostral retrosplenial level. A caveat comes from studies with mice showing how neurotoxic lesions of the cingulate cortices can have different effects to cortical lesions that include the cingulum (Meunier and Destrade, 1988, 1997).

Table 1 demonstrates how fornix lesions produce more severe spatial memory deficits than cingulum bundle lesions. These differences include a striking dissociation for an automated test of spatial working memory (delayed nonmatching-to-position). Neither cingulum bundle nor cingulate cortex lesions have any apparent effect on this task. In contrast, both fornix and anterior thalamic lesions, as well as hippocampal and medial prefrontal lesions (Aggleton et al., 1992, 1995), persistently impair this task. This dissociation shows that for some classes of spatial memory the dominant interactions are hippocampal – thalamic – frontal, but using routes that are not cingulum dependent.

**Fig. 7:**
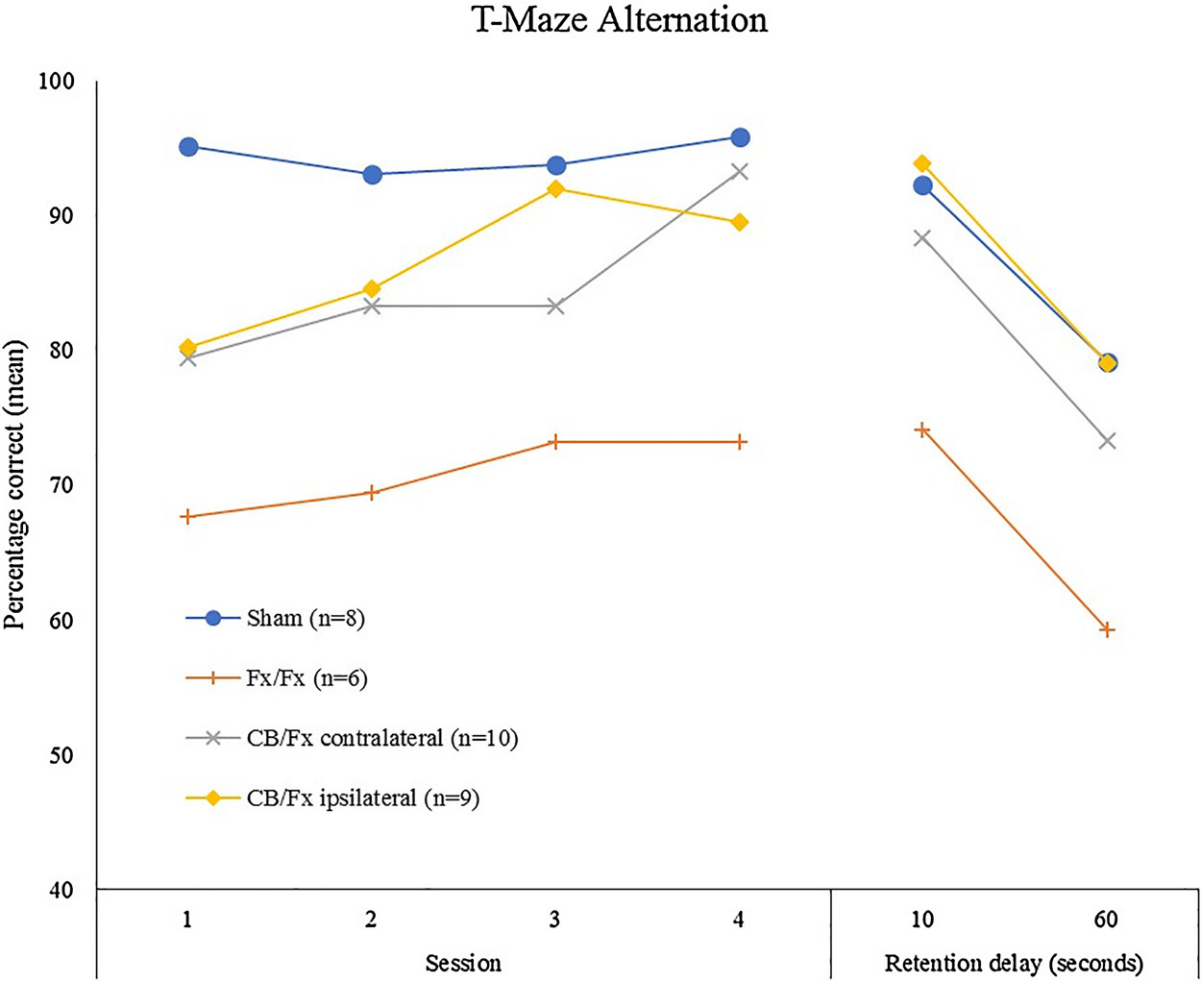
Performance of rats with unilateral cingulum bundle and fornix lesions in opposite hemispheres (CB/FX contralateral) on T-maze alternation (chance performance 50%; unpublished findings). Later sessions contained a mixture of 10s (as in training) and 60s retention intervals between sample and choice runs. Comparison performance is shown for rats with bilateral fornix lesions (Fx/Fx), unilateral cingulum bundle and fornix lesions in the same hemisphere (CB/Fx ipsilateral), and surgical controls (Sham).

These fornix and cingulum bundle lesion differences are notable as both tracts are serially linked within ‘Papez circuit’ (Fig. 2), which is assumed to be vital for normal cognition (Aggleton and Brown, 1999; Rolls, 2015). To test whether these two tracts share common information required for spatial memory, crossed lesions were studied, i.e., a unilateral fornix lesion in one hemisphere combined with a unilateral cingulum bundle lesion in the contralateral hemisphere. The cingulum lesions were made at two anterior-posterior levels to enhance the extent of disconnection (see Warburton et al., 1998). These disconnection surgeries were compared with unilateral lesions in the same hemisphere (i.e., ipsilateral), as well as bilateral fornix lesions. Despite the cingulum bundle lesions being made at two levels (one retrosplenial, one close to the genu), the contralateral cingulum-fornix surgery had little or no apparent effect on T-maze alternation or working memory in a radial-arm maze (Fig. 7, unpublished data). In contrast, bilateral fornix lesions severely disrupted both tasks.

The lack of a disconnection effect indicates that information in the cingulum bundle either duplicates or is qualitatively different from that in the fornix. One potential example of the latter concerns landmark and head-direction information within retrosplenial cortex (Jacob et al., 2016), which presumably reflect its cingulum connections. Another issue is that the pathways between the hippocampal formation, anterior thalamic nuclei, and retrosplenial cortex might contain sufficient crossed projections to nullify the impact of the disconnection (Mathiasen et al., 2017). While this concern is difficult to disprove, a complex disconnection procedure (Dumont et al., 2010) found evidence that anterior thalamic lesions and retrosplenial cortex lesions have additive effects, implying different contributions to hippocampal function. These additive effects (Dumont et al., 2010) support the results of the cingulum/fornix disconnection study (Fig. 7) by emphasising differences in the functional contributions of these two major tracts to spatial learning and memory. Nevertheless, these studies largely leave unanswered why cingulum bundle lesions often have such slight effects on spatial learning given the significance of this tract for both anterior thalamic and hippocampal fibres.

Finally, there is evidence that connections within the rostral cingulum bundle support attentional processes. Lesions of the anterior cingulate cortex disrupt performance on the 5-Choice Serial Reaction Time Task (5-CSRTT) (Dalley et al., 2004), while related studies show how cholinergic activity is intrinsic to 5-CSRTT performance (Dalley et al., 2004). These findings implicate the cingulum given its role in providing cholinergic inputs to medial frontal and cingulate areas. Related evidence comes from the disruptive effects of medial frontal lesions when switching between stimulus classes (‘extra-dimensional shift’) during serial discriminations (Birrell and Brown, 2000). Similar lesions also affect effort-based decision making (Walton et al., 2002). These studies highlight cognitive functions that have yet to be examined following cingulum bundle interventions in rats. Likewise, beyond studies of pain, emotional processes have not been studied following cingulum bundle manipulations. Further, evidence that anterior cingulate lesions can decrease social responsiveness and the memory of social stimuli in rats (Rudebeck et al., 2007) points to the likely involvement of the bundle.

### 3.2 Nonhuman primates (macaque monkeys)

Selective cingulum bundle lesions have not been investigated in nonhuman primates. For this reason, surgical lesions in those adjacent cingulate areas that contribute many fibres to the tract, are considered. It can be assumed that, unless otherwise stated, these surgeries consistently involved the bundle. We first consider lesions encompassing both the anterior and posterior cingulate cortices (including retrosplenial cortex), as they will produce the greatest loss of cingulum fibres.

Inspired by Papez’ model (1937; see Fig. 2), many initial studies focussed on emotion. However, the effects of cingulate gyrus lesions on social and affective behaviour appear inconsistent. Extensive cingulate resections (anterior plus posterior cortices) had little apparent effect on social group affinity or individual aspects of affective behaviour in free ranging rhesus monkeys (Franzen and Myers et al., 1973; Myers et al., 1973). Likewise, monkeys with anterior cingulate lesions seemed to show no consistent, overt changes in social behaviour, activity, or vocalisation (Pribram and Fulton, 1954). Similarly, galvanic skin responses to novel and familiar tones appeared unaffected after such lesions (Kimble et al., 1965).

In contrast, other studies suggest that anterior cingulate lesions cause monkeys to become more docile and less shy of humans (Anand et al., 1957; Glees et al., 1950; Ward, 1948). It was also reported that large medial frontal lesions around the genu (involving areas 24, 32, 25) reduce social interactions and vocalisations (Hadland et al., 2003). Other anterior cingulate lesions around the genu reduced responsiveness to social cues, implying that this region helps evaluate such stimuli (Rudebeck et al., 2006). In contrast, orbital frontal lesions (which spare the cingulum) additionally disrupt emotional responsiveness to non-social cues, e.g., to a snake (Rushworth et al., 2007).

Regarding other functions, extensive cingulate resections above corpus callosum, which again involved the cingulum bundle, had no apparent effect on T-maze alternation (Murray et al., 1989) or the ability to learn a scene discrimination task thought to capture elements of episodic memory (Parker and Gaffan, 1997), yet both tasks are hippocampal sensitive (Gaffan, 1994; Murray et al., 1989). Related results include how anterior cingulate lesions can spare spatial delayed response (Pribram and Fulton, 1954), although more extensive anterior cingulate removals above and below the genu (Meunier et al., 1997) may cause mild deficits on spatial reversal learning, delayed response, and object recognition memory. In other reports, delayed alternation was spared, even though the anterior cingulate gyrus lesions again consistently involved the cingulum bundle (Rushworth et al., 2003). Taken together, these findings indicate a preservation of working memory *per se* following dorsal cingulum bundle damage (Meunier et al., 1997; Murray et al., 1989; Pribram and Fulton., 1954).

While rostral cingulate gyrus lesions centred around the genu and involving the cingulum failed to affect a visual discrimination learning task, they did impair a conditional task in which different rewards were linked with different actions (Hadland et al., 2003). From this and related findings (Kennerley et al., 2006; Rushworth et al., 2004), it was proposed that the macaque anterior cingulate cortex helps in monitoring and reacting to particular forms of conflict, but that this executive control function may not always be revealed by selective lesions as this function is shared with other frontal areas (Mansouri et al., 2017). Related evidence from monkeys performing a Wisconsin Card Sorting Test (WCST) again points to a role for the anterior cingulate cortex in representing error likelihoods and adjusting behavioural patterns following an error (Buckley et al., 2009; Kuwabara et al., 2014).

Lesions in posterior cingulate/retrosplenial cortices should induce different impairments, reflecting their greater interaction with hippocampal and parahippocampal regions. In one of the very few such studies, removal of the supracallosal parts of areas 29, 30 and 23 (Mansouri et al., 2015) had no apparent effect on a computerised version of the WCST, in contrast to the effects of anterior cingulate damage (Buckley et al., 2009). (It is likely that the retrosplenial surgeries partially involved the cingulum). Aspiration lesions centred on retrosplenial cortex also spared the acquisition of scene discriminations (Buckley and Mitchell, 2016; see also Parker and Gaffan, 1997), but did disrupt the retention of these discriminations (Buckley and Mitchell, 2016). This retention deficit is informative as both anterior thalamic nuclei and fornix lesions impair the initial learning of these same problems (Gaffan, 1994; Parker and Gaffan, 1997). Given the close interconnectivity between the anterior thalamic nuclei, hippocampus, and posterior cingulate/retrosplenial cortices, points of similarity and difference following lesions are especially notable.

Because studies have not targeted the cingulum itself, but often included it within larger lesions, some of the most telling findings are null results. Despite the presumed disconnections caused by the various surgeries, the impact on social behaviour, emotion, memory, and executive control is often slight, implying that damage more confined to the bundle might be even less disruptive. Meanwhile, there is a gap in our knowledge concerning lesions of the parahippocampal cingulum, but its location immediately adjacent to the presubiculum, parasubiculum, and angular bundle, makes selective interventions very challenging.

### 3.3 Humans - cingulotomy

Following the introduction of prefrontal lobotomy for mental illness (Moniz and de Almeida Lima, 1935), there was a perceived need to disrupt more specific connections (Turner, 1973). As a key structure in Papez’s (1937) circuit for emotion, the anterior cingulum bundle became a target. In anterior cingulotomies, bilateral lesions are made in the white matter underlying the anterior cingulate cortex (Fulton, 1951). From 1962, stereotactic methods were employed (Corkin, 1980). The advent of computerised tomography and subsequent MR imaging, confirmed that although anterior cingulotomies do compromise the cingulum (Steele et al., 2008; Turner., 1973), they cause additional cortical damage, principally in area 24 (Spangler et al., 1996; Steele et al., 2008).

Difficulties in interpreting this clinical literature include the lack of formal post-operative assessments in early studies, the frequent absence of appropriate controls, and the failure to test blind to surgical treatment. This section, therefore, incorporates more rigorous, recent research, which also benefits from better visualisation of the surgery. Using the latter information, Heilbronner and Haber (2014) concluded that typical cingulotomies compromise a great many fibres in the supracallosal cingulum bundle, involving a wide variety of cortical and subcortical sites, including both amygdala and anterior thalamic fibres.

The first anterior cingulotomies (Whitty et al., 1952) were to treat schizophrenia. As no lasting benefits occurred (Corkin, 1980), cingulotomy for psychotic patients was generally abandoned (Ballantine et al., 1967). More encouraging results were, however, noted in an obsessional patient and in one with anxiety disorder (Whitty et al., 1952), prompting a switch to these conditions. A prospective analysis of 198 cases with bilateral cingulum bundle lesions (Ballantine et al., 1987) pointed to lasting improvements in approximately half of those treated for obsessive compulsive disorders (OCD) (see also Hay et al., 1993; Jung et al., 2006; but see Richter et al., 2004), with slightly better outcomes for anxiety disorders and affective disorders, including depression (Ballantine et al., 1987; Shields et al., 2008). Other analyses have echoed this pattern of results (Corkin, 1980; Feldman et al., 2001; Spangler et al., 1996; Steele et al., 2008), including those using standardised assessments, such as pre and postoperative score comparisons on the Beck Depression Inventory (Shields et al., 2008). Importantly, the beneficial impact on OCD on those who respond has been shown to persist for at least two years (Jung et al., 2006)

The apparent ability of anterior cingulotomy to provide some level of relief for a range of psychiatric illnesses is initially striking (Feldman et al., 2001; Linden, 2014). However, the nature of the improvements reveals some similarities across patient groups. Anxiety, OCD, and bipolar depression patients are sometimes jointly seen as exhibiting less anxiety, depression, hostility and obsessional thinking post-cingulotomy (Brown and Lighthill, 1968), of which a common feature appears to be a lessening in attention to negative thoughts, anxieties, and tension (Cohen et al., 2001). Indeed, Tow and Whitty (1953) reported that negative emotions and obsessional thoughts still occurred post-cingulotomy, but that they ‘no longer bothered’ their patients. Related studies have described positive affect after surgery as shallower, while motivation is depressed, but not to a clinical degree (Hay et al., 1993; Tow and Whitty, 1953; Whitty et al., 1952; Wilson and Chang, 1974).

Neurosurgeons also targeted the anterior cingulum for the treatment of chronic pain. Patients are reported as continuing to experience pain but to perceive it as less distressing or less worrying (Foltz and White, 1962; Corkin and Hebben, 1981), with over 50% having appreciable relief (Rawlings et al., 1992; Sharim and Pouratian, 2016). Further, it may be those patients with anxiety or depression who benefit most (Foltz and White, 1962). Cohen et al. (2001) attempted to formally investigate these changes, evaluating emotional and personality characteristics of 18 patients undergoing cingulotomy for chronic pain using standardised psychological tests. A year after surgery, the cingulotomy patients, compared to controls, showed the greater improvement on tension and anger scales (Cohen et al., 2001). Meanwhile, no alterations were observed in measures of self-perceived energy and emotional vibrancy (Cohen et al., 2001). Whilst these results suggest that subjective positive emotions are less affected by cingulotomy, it should be noted that behavioural passivity and apathy remain frequently reported by families of patients (Cohen et al., 1999b, 2001).

Concerning apathy, it is the case that limbic-frontal-subcortical circuits are frequently implicated in the pathophysiology of this condition in clinical populations (van Reekum et al., 2005; Kos et al., 2016). Across differing underlying pathologies and neuroimaging methods, the anterior cingulate cortex, orbital frontal cortex, and medial thalamus exhibit some of the most robust changes associated with apathy (Le Heron et al., 2018). In addition, apathy is a well-recognized feature of strokes affecting similar portions of the medial frontal cortex (Kang & Kim, 2008; Le Heron et al., 2018) and the medial and anterior thalamus (Carrera & Bogousslavsky, 2006; Krause et al., 2012; Serra et al., 2013) respectively. The interconnections between these structures, therefore, take prominence in many circuit level explanations of apathy (van Reekum et al., 2005; Kos et al., 2016; Le Heron et al., 2018) and the likely disruption of many of these fibres in anterior cingulotomy may explain the prevalence of apathetic characteristics in this patient group. It is notable that spontaneous response initiation and generation were both reduced in a study of 12 cingulotomy patients when tested over a year post-surgery (Cohen et al., 1999b).

It is striking that surprisingly few lasting cognitive disturbances are described after anterior cingulotomies. Both informal reports (Tow and Whitty, 1953; Turner, 1973) and formal assessments (Cohen et al., 1999a,b; Corkin, 1980) often indicate preserved cognition, although transient confusion, disorientation, and memory loss can occur (Dougherty et al., 2003; Sharim and Pouratian, 2016; Whitty and Lewin, 1960). Meanwhile, IQ measures typically remain the same or even slightly improve post-surgery (Ballantine et al., 1967, 1987; Brown and Lighthill, 1968; Cohen et al., 1999a,b; Corkin, 1980; Fedio and Ommaya, 1970; Kim et al., 2003; Steele et al., 2008), with any improvement seen as an increased ability to deal with disease burden (Ballantine et al., 1967).

In an extensive study of cingulotomy for pain or depression, Corkin (1980) found no deficits on multiple tests of frontal-lobe function including fluency tests, delayed-alternation, maze tracing and the WCST for 34 patients tested both before and over one year after surgery. In contrast, a smaller group of cingulotomy patients (n = 14) treated for OCD showed impaired WCST performance (Kim et al., 2003). Likewise, in eight patients surgically treated for depression, performance on tests of executive function and memory, including block design, verbal fluency and digit span, appeared unaltered (Steele et al., 2008). In addition, there were improvements in paired-associate learning and spatial working memory. The same study (Steele et al., 2008) also found no deficits on the Trail Making Test, which taxes visual attention and task switching, although borderline deficits had previously been reported in a larger cohort of 18 cases (Cohen et al., 1999b).

Despite its many limitations, the consistent lack of a deficit on the Wechsler Memory Scale after anterior cingulotomy is striking (Fedio and Ommaya, 1970; Corkin, 1980; Cohen et al., 199b). Other formal tests of memory, including recall of the Rey-Taylor complex, also show preserved performance (Corkin, 1980; Jung et al., 2006). Similar studies have again found no long term changes in the performance of other memory tasks, including the Hopkins Verbal Learning Test (Kim et al., 2003; Jung et al., 2006). Finally, while stimulation of the left cingulum bundle in five patients disrupted a verbal working memory task, subsequent cingulotomy for pain relief did not affect levels of performance (Fedio and Ommaya, 1970).

Other evidence points, however, to post-surgical deficits in focused and sustained attention, as well as mild changes in executive function (Cohen et al., 1999a,b). Difficulties have been reported for self-initiated responding, e.g., spontaneous verbal utterances, leading to reduced behavioural spontaneity (Cohen et al., 1999a,b). Frontal-type deficits have also been seen in a Stroop Interference task, with borderline deficits on a Go/No-Go executive task (Cohen et al., 1999b). Post-surgical deficits were also found in older patients on a test of visual perception (Thurstone’s Hidden Figures) that potentially taxes executive functions (Corkin, 1979). Furthermore, OCD sufferers after cingulotomy showed poorer WCST performance on several measures (Kim et al., 2003; but see Corkin, 1980, Cohen et al., 1999b), consistent with a loss of executive control. Together, these observations point to an involvement of the anterior cingulate/cingulum area in high-level processing and selection, though the lack of consistent deficits points to contributions from other pathways.

One key issue concerns the location and extent of the most effective cingulotomies. While some reports imply that extending the cingulum lesion with a second surgery can give a more favourable outcome (Spangler et al., 1996; but see Dougherty et al,. 2003), a study of major depression found that smaller lesion volumes were associated with better results (Steele et al., 2008). This same study of 8 cases found that lesions placed a little behind the genu were associated with better outcomes than those more caudal, at mid cingulate levels (Steele et al., 2008; see also Corkin, 1980; Richter et al., 2004). This placement is interesting as it should affect medial prefrontal, anterior cingulate, and amygdala interactions thought to be involved in emotion and cognitive processing (Heilbronner and Haber, 2014; Steele and Lawrie, 2004). Indeed, neuroimaging studies have consistently found this same region to function abnormally in disorders such as major depression (Drevets, 2000), while hypermetabolism in the subgenual cingulate and prefrontal cortex may predict favourable responses to anterior cingulotomies in this disorder (Dougherty et al., 2003).

Posterior cingulotomies have rarely been performed. An exception concerned the attempted treatment of chronic aggression in extreme cases of paranoia or personality disorder in five cases (Turner, 1973). The procedure involved the posterior cingulate gyri above the splenium, as well as the bundle. It is not, however, possible to determine the extent of cingulum bundle involvement. Post-operative reductions in aggression were reported, while memory was thought to be unaffected, although formal assessments were lacking (Turner, 1973).

Overall, many of the descriptions of cingulotomy appear consistent with the anterior cingulate and adjacent cingulum bundle having a role in the integration of visceral, and affective processes (Dalgleish, 2004), e.g., causing less attention to negative states. Reflecting these roles, cingulate projections may be involved in the maintenance and cortical integration of information from limbic structures, which includes conflict between the current status and perceived indicators of change (Mansouri et al., 2017; Rushworth et al., 2004). The cingulum can also play a related role in amplifying or attenuating emotional responses to pain signals (Cohen et al., 1999b). The discovery of selective deficits in attention and cognitive control also points to a contribution to executive tasks (Cohen et al., 1999a,b; Janer and Pardo, 1991). Nevertheless, it is the apparent lack of more overt cognitive changes after anterior cingulotomy that is often most striking, a finding that echoes the earlier review of monkey research (Section 3.2).

## 4 Diffusion MRI (dMRI) studies of the human cingulum bundle

### 4.1 Introduction

Rather than studying intentional cingulum bundle damage (cingulotomies), brain imaging allows the non-invasive investigation of individual differences or changes in cingulum microstructure by means of correlational analysis between DTI indices and cognition and clinical symptoms or via between group comparisons. Based on its changing constitution (Fig. 5), it can be predicted that anterior (dorsal) cingulum characteristics will correlate with attention and executive functions (i.e., frontal processes) while the parahippocampal (temporal) cingulum will be more closely linked to learning and episodic memory (see Section 4.2). Like most white matter tracts, the cingulum bundle changes over the lifespan. Diffusion imaging studies have revealed an extended period of cingulum maturation through adolescence and beyond, often not reaching its adult characteristics until the mid twenties or later (Lebel and Beaulieu, 2011; Lebel et al., 2012). Remarkably, the mean age to reach peak fractional anisotropy was found to be 42 years (Lebel et al., 2012), making the cingulum one of the last major tracts to mature by this measure. This lengthy period of transition and change has particular implications for cognitive and emotional skills that develop through adolescence and beyond.

The cingulum is affected in many neurological conditions, including multiple sclerosis, Parkinson’s disease, the behavioural variant of frontemporal dementia (Mahoney et al., 2014), Mild Cognitive Impairment (MCI), and Alzheimer’s disease (AD). This review considers amnestic MCI and AD (Section 4.4), prefaced by normal aging (Section 4.3). The final sections concern psychiatric states. Attention is focussed on those relatively common states for which there is repeated evidence of cingulum change. These conditions are schizophrenia, attention deficit hyperactivity disorder (ADHD), depression, post-traumatic stress disorder (PTSD), obsessive compulsive disorder (OCD), and autism spectrum disorder (ASD). These sections begin with those conditions where there is additional evidence from cingulotomies (Section 3.3). Meanwhile, Table 2 highlights the status of specific subdivisions of the bundle in these various psychiatric conditions, alongside correlations with psychometric data. The commonest reported changes are reduced FA and increased diffusivity, both of which are though to reflect a disruption of white matter integrity.

**Table 2:** Examples of diffusion MRI studies that have reported cingulum bundle changes in schizophrenia, attention deficit hyperactivity disorder (ADHD), depression (including major depressive disorder, MDD), post-traumatic stress disorder (PTSD), obsessive compulsive disorder (OCD), and autism spectrum disorder (ASD). The columns show which portion of the cingulum appeared abnormal and provide neuropsychological correlations. Relevant meta-analyses are in the right column. Other abbreviations: FA, fractional anisotropy; GFA, global FA; MD, mean diffusivity; RD, radial diffusivity. Reductions in FA and increases in diffusivity are usually seen as evidence of a loss of white matter integrity.

Clinical group	Cingulum subsection	Structural change	Supporting research	Neuropsychological correlations	Meta-analysis conclusions	
Schizophrenia	Dorsal	FA -	Takei et al., 2009; Kubicki et al., 2003	Lower FA in the left dorsal cingulum correlated with poorer performance on the Wisconsin Card Sorting Test (Kubicki et al., 2003).	Moderate to high quality evidence exists of a reduction in white matter density and FA in the cingulum in schizophrenia (Shepherd et al., 2012).	
	Dorsal	MD +	Takei et al., 2009	Higher MD in dorsal cingulum correlated with a longer reaction time on the Stroop Test.		
	Dorsal, pregenual anterior	FA -	Takei et al., 2009			
	Dorsal, anterior (RH)	FA -	Sun et al., 2003; Wang et al., 2004; Fujiwara et al., 2007a ; Fujiwara et al., 2007b; Hao et al., 2006 ; Whitford et al., 2014	Lower FA in the right dorsal anterior cingulum correlated with patient scores of hallucinations and delusions (Whitford et al., 2014).		
	Dorsal, anterior (LH)	FA -	Sun et al., 2003; Wang et al., 2004; Fujiwara et al., 2007a; Fujiwara et al., 2007b; Mitelman et al., 2007			
	Dorsal, posterior (RH)	FA -	Fujiwara et al., 2007a			
	Dorsal, posterior (LH)	FA -	Fujiwara et al., 2007a, Mitelman et al., 2007			
	Ventral (RH)	FA -	Whitford et al., 2014	Lower FA in the right ventral cingulum correlated with patient scores of affective flattening and anhedonia/associability		
ADHD	Dorsal, anterior (RH)	FA -	Makris et al., 2007; Konrad et al., 2010			Evidence exists of disturbed white matter integrity in the cingulum in ADHD, but it is not one of the structures most reliably reported to be effected (van Ewijk et al., 2012).
	Dorsal, posterior	FA +	Svatkova et al., 2016			
Depression (bipolar)	Dorsal (RH)	MD +	Benedetti et al., 2011			Evidence of disturbed white matter integrity in the cingulum is mixed in depressive clinical populations. Stronger evidence exists of microstructure alteration in 'at risk' groups (Bracht et al., 2015).
	Dorsal (RH)	RD +	Benedetti et al., 2011			
	Dorsal, anterior	FA -	Wang et al., 2008			
	Dorsal, posterior (LH)	FA -	Wise et al., 2016			
Depression (MDD)	Dorsal	FA -	de Diego-Adelino et al., 2014			
	Dorsal, subgenual anterior	FA -	Cullen et al., 2010			
PTSD	Dorsal	FA +	Kennis et al., 2015	Greater FA in the dorsal cingulum correlated with symptom severity and persistence (Kennis et al., 2015; Kennis et al., 2017).		A small meta-analysis concluded there is preliminary evidence of group differences in cingulum integrity in PTSD. Evidence indicates increases and decreases in FA in different sections of the cingulum (Daniels et al., 2013).
	Dorsal (LH)	FA -	Kim et al., 2006; Sanjuan et al., 2013			
	Dorsal (RH)	FA -	Sanjuan et al., 2013			
	Dorsal, anterior	FA -	Zhang et al., 2011			
OCD	Dorsal (LH)	FA +	Cannistraro et al., 2007; Gruner et al., 2012	Greater FA in the left dorsal cingulum correlated with better performance in response inhibition and cognitive control measures; the Stroop Test and the Trails Making Test (Gruner et al., 2012).		1. There is robust evidence of increased white matter volume and decreased FA in anterior midline tracts (including the cingulum) in OCD (Radua et al., 2014). 2. There is evidence FA is typically reduced in the cingulum in adults and increased in paediatric and adolescent samples (Koch et al., 2014).
	Dorsal (RH)	FA -	Cannistraro et al., 2007			
	Dorsal, anterior (LH)	GFA -	Chiu et al., 2011	GFA in left anterior cingulum correlated with higher scores in measures of obsession.		
	Dorsal (RH)	MD -	Lochner et al., 2012	MD in the right body of the dorsal cingulum negatively correlated with scores in measures of anxiety and depression.		
	Dorsal, anterior (LH)	MD -	Lochner et al., 2012	MD in the left anterior cingulum correlated with scores on an obsessive compulsive scale.		
	Ventral (LH)	FA -	Fan et al., 2016			
ASD	Dorsal	FA -	Ikuta et al., 2014; Shukla et al., 2011	Reduced FA in the cingulum correlated with poorer behavioural regulation scores (Ikuta et al., 2014).		1. There is evidence of cingulum microstructure changes in autism, most consistently reduced FA and/or increased MD in the anterior cingulum (Travers et al., 2012). 2. Combining datasets from five studies found no evidence to support a significant difference in cingulum FA between autistic subjects and typically developing controls (Aoki et al., 2013).
	Dorsal	MD +	Shukla et al., 2011			
	Dorsal	RD +	Shukla et al., 2011			
	Dorsal, anterior	FA -	Jou et al., 2011			

Throughout, it is important to remember that such dMRI analyses are only correlative and not causal in nature. Indeed, the growing realisation that experience can alter white matter microstructure (Metzler-Baddeley et al., 2017; (McKenzie et al., 2014) Zatorre et al., 2012) highlights the problems of separating cause from effect. It must also be remembered that in none of the clinical conditions described is pathology restricted to the cingulum bundle.

### 4.2 Executive function and memory

There is accumulating evidence that the cingulum bundle, notably its dorsal/anterior portions, mediates performance in ‘frontal’ tests of cognitive control and executive function. A recent study by Bettcher et al. (2016) employed latent variable modelling to investigate the relative contribution of individual differences in prefrontal grey matter volume and white matter microstructure of the dorsal cingulum, the corpus callosum and the superior longitudinal fasciculus to performance variations in executive function components (shifting/inhibition, updating/working memory and processing speed) in a group of 202 community dwelling adults. They found individual FA differences in the dorsal cingulum to contribute independently to all executive functions whilst prefrontal cortex grey matter volume did not independently predict executive performance. These results are consistent with a number of previous reports of correlations between FA metrics in the cingulum and working memory, attention and executive functions (Charlton et al., 2010; Kantarci et al., 2011; Metzler-Baddeley et al., 2012a,b; Takahashi et al., 2010; Yamamoto et al., 2015; Chiang et al., 2016).

For instance, Kantarci et al. (2011) employed an ROI approach to study the contribution of anterior and posterior cingulum FA and MD to attention/executive, language, memory, and visuo-spatial function in a group of 220 cognitive healthy older adults. They found FA differences in the anterior cingulum to correlate with differences in attention/executive and memory performance, while FA in the posterior dorsal cingulum appeared to contribute to all four cognitive domains. Another study (Metzler-Baddeley et al., 2012b) used DTI tractography to reconstruct anterior, middle, posterior and parahippocampal cingulum portions of the cingulum and found individual FA differences in the anterior and posterior cingulum portion but not the middle or the parahippocampal portion to correlate with executive function tasks (Category Fluency and Stroop Test). This overall pattern is continued in clinical brain imaging studies as correlations between executive functions and cingulum white matter properties have been reported in depression (with DTI in Schermuly et al., 2010), in bipolar disorder (with DTI in Poletti et al., 2015; with Positron Emission Tomography in Li et al., 2012), and schizophrenia (with DTI in Kubicki et al., 2009; Takei et al., 2009; with VBA in Spalletta et al., 2014; see Table 2). It is apparent that while this relationship with executive function echoes that reported in some studies of cingulotomy (e.g., Cohen et al., 1999a,b), the dMRI results suggest tract involvement in a wider range of attributes and tests.

Much of the relevant imaging literature on long-term memory concerns clinical conditions that affect the temporal lobes, e.g., Section 4.4. Nevertheless, correlations between episodic memory and left parahippocampal cingulum FA that were independent of hippocampal volume were also found in non-dementing elderly adults (Ezzati et al., 2016). However, such cingulum associations with episodic memory are not always observed in healthy older individuals (Metzler-Baddeley et al., 2011) nor are they always confined to its parahippocampal subdivision (e.g., Kantarci et al., 2011). Compared with other limbic white matter pathways, correlations between white matter microstructure and episodic memory in healthy populations are much more reliably found for the fornix than the cingulum (e.g., Douet and Chang, 2015; Metzler-Baddeley et al., 2011; Rudebeck et al., 2009).

In contrast to the evidence from healthy populations, correlations between cingulum microstructure and memory are more often described within clinical groups, e.g., in cerebral small vessel disease (van der Holst et al., 2013), mild traumatic injury (Wu et al., 2010), Mild Cognitive Impairment (e.g., Metzler-Baddeley et al., 2012a, b, for meta-analysis see Yu et al., 2017) and Alzheimer’s disease (e.g., Kantarci et al., 2017). More specific associations between memory performance and FA and MD metrics of the parahippocampal cingulum were found for both immediate and delayed visuospatial memory in a group of school-aged survivors of neonatal complications (Schiller et al., 2015, 2017a), for verbal memory in temporal lobe epileptics, (McDonald et al., 2008), as well as for episodic memory and RD and AD metrics in patients with multiple sclerosis (Koenig et al., 2015). Other evidence implicating the parahippocampal cingulum in memory comes from studies of MCI and AD (Section 4.4). Consequently, while there is an overall shift from genual parts of the cingulum (executive function) to parahippocampal parts (memory), this shift appears to be gradual in nature and the latter association appears more robust in some clinical populations.

### 4.3 Healthy aging of the cingulum bundle

A number of studies suggest that aging does not affect the cingulum uniformly but that region-specific effects can be observed. Some of the evidence is consistent with an age-related gradient along the long axis of the cingulum, in which frontal parts of the bundle appear most affected (Catheline et al., 2010; Mårtensson et al., 2018; Sibilia et al., 2017; Yoon et al., 2008; see for review Chua et al., 2008). For example, older versus younger adults exhibited lower FA and AD as well as increased RD in the left subgenual cingulum, but not in the retrosplenial or parahippocampal cingulum (Sibilia et al., 2017). Similarly, Jang et al. (2016) investigated age differences across five portions of the cingulum (anterior, anterior superior, posterior superior, posterior and inferior cingulum) in 90 participants between 20 and 78 years of age. They reported decreased FA in anterior and anterior superior portions of the cingulum in older versus younger adults. However, as age-related changes were also observed in the number of reconstructed fibres in the parahippocampal cingulum they proposed that aging may affect both ends of the cingulum across the lifespan (see also Chua et al., 2008).

Addressing this prediction, Mårtensson et al., 2018 investigated age-related differences in anterior, posterior and inferior cingulum portions in 257 healthy individuals between 13 and 84 years of age. They found age-related FA differences in the anterior and posterior but not in the inferior portions of the cingulum whilst RD, AD and MD showed also effects in the inferior portion. In the posterior cingulum, FA reached a maximum in early adulthood up to the age of 40 and then declined, whilst RD was lowest at midlife and increased thereafter with age. In the anterior portion of the cingulum, RD values increased from the age of 28. Overall this study demonstrated that age-related individual differences varied between and within individual tracts. This pattern of changes may reflect age-related differences in biological properties of white matter microstructure in different cingulum sections but could also arise due to differences in the fibre complexity between different portions.

### 4.4 Mild cognitive impairment (MCI) and Alzheimer’s disease (AD)

Amnestic MCI is often considered a prodromal condition for AD. A well-replicated finding concerns microstructural changes in the posterior and parahippocampal cingulum associated with both MCI and AD (Bozzali et al., 2012; Choo et al., 2010; Metzler-Baddeley et al., 2012a; Remy et al., 2015; Wang et al., 2017; Zhang et al., 2007; Zhuang et al., 2012; 2013, for review see Chua et al., 2008; Yu et al., 2017).

Yu et al. (2017) conducted an activation likelihood estimation meta-analysis of 77 studies into microstructural changes in amnestic MCI compared with healthy controls either in medial temporal lobe white matter pathways, including the parahippocampal cingulum, or across the white matter of the whole brain. Studies that employed ROI analysis, including tractography, showed consistent reductions in FA and increases in MD in the fornix, the parahippocampal cingulum, and the uncinate fasciculus, whilst whole brain analysis study based on TBSS or VBA demonstrated significant FA reductions in the posterior corona radiata. For instance, one of the included studies (Choo et al., 2010) averaged FA and MD values over 3 × 3 x 3 mm cubric voxels placed over middle, posterior and parahippocampal ROIs of the cingulum. This study reported reduced FA in the parahippocampal cingulum in both MCI and Alzheimer’s patients, with additional FA changes in the posterior retrosplenial cingulum in the Alzheimer’s group (Choo et al., 2010; see also Ito et al., 2015). This pattern of white matter differences is consistent with the well-established grey matter loss in parahippocampal, hippocampal, and posterior cingulate regions in Alzheimer’s disease (Choo et al., 2010; Chua et al., 2008). However, given the size of the ROI voxels (see Fig. 1 Choo et al., 2010), it is likely that average FA and MD metrics were biased by partial volume effects from cerebrospinal fluid (CSF) contamination due to patients’ brain atrophy. As CSF based partial volume effects are known to result in artificial FA reductions and MD increases (Alexander et al., 2001; Vos et al., 2011; Metzler-Baddeley et al., 2012a), differences between patients and controls may be overestimated and could potentially reflect atrophy rather than intrinsic white matter microstructural changes.

Metzler-Baddeley and co-workers (Metzler-Baddeley et al., 2011; Metzler-Baddeley et al., 2012b, c; Christiansen et al., 2016) addressed the problem of potential CSF partial volume biases in DTI metrics in MCI patients by applying the bi-tensor Free Water Elimination (FWE) model that fits an isotropic and an anisotropic compartment to the dMRI data and allows for the correction of free water contamination in DTI metrics (Pasternak et al., 2009). The authors compared corrected mean FA, MD, RD and AD metrics for the parahippocampal cingulum, the fornix, and uncinate fasciculus between 25 MCI patients and 20 matched controls (Metzler-Baddeley et al., 2012b), with the cortico-spinal tract as a comparison pathway. In MCI patients, FWE corrected RD was increased in all limbic pathways and AD was increased in the uncinate fasciculus and in left but not right parahippocampal cingulum. No group differences in any metrics were present for the cortico-spinal control tract. Furthermore, and in contrast to MCI studies without FWE correction (see Yu et al., 2017), Metzler-Baddeley et al. (2012a, b) did not find any FA reductions or increases in MD, but rather observed a trend for increased FA in left and right parahippocampal cinguli in MCI. Metzler-Baddeley also investigated correlations between individual differences in white matter microstructure and performance in episodic memory (Metzler-Baddeley et al., 2011; Metzler-Baddeley et al., 2012a,b; Ray et al., 2015) and executive function tasks (Metzler-Baddeley et al., 2012a). Whilst healthy controls showed robust correlations between performance in episodic memory tasks (free recall and recognition) and FA in the fornix but not the parahippocampal cingulum, MCI patients showed correlations between recognition memory and microstructure in both pathways (Metzler-Baddeley et al., 2012a,b). Similarly, performance in executive functions, which predominately correlated with anterior cingulum portions in controls, was also associated with parahippocampal microstructure in the patient group (Metzler-Baddeley et al., 2012b).

A follow-up study (Ray et al., 2015) then demonstrated that the shift from correlations between memory and the fornix to correlations between memory and the parahippocampal cingulum was largest for MCI patients with better memory performance and larger basal forebrain volume. The latter provides an estimate of relative atrophy in the frontal cholinergic system. Together this pattern of results suggests that in the presence of fornix impairments, episodic memory can be supported by the parahippocampal cingulum and that such compensatory processes may depend on the integrity of cholinergic innervation from the basal forebrain to the limbic system and, hence, may contribute to “cognitive reserve” in MCI (Ray et al., 2015). In addition, this example highlights how dMRI processing choices, i.e. with or without FWE correction can impact on the observed pattern of results (see Yu et al., 2017).

Further evidence of a relationship between cingulum microstructure and disease severity comes from a study that found correlations between Alzheimer’s disease patients’ Mini-Mental State Examination (MMSE) scores and MD in the posterior cingulum (Nakata et al., 2009). Likewise, the severity of Alzheimer’s disease can correlate with parahippocampal cingulum FA (Kantarci et al., 2017). In addition, MD increases in the right parahippocampal cingulum were found to correlate with disease-related cortical thinning in parahippocampal grey matter regions and with episodic memory impairments in amnestic MCI (Wang et al., 2017). Finally, MCI patients showed reduced FA in the retrosplenial cingulum, but not the anterior cingulum, as well as inter-individual differences in retrosplenial cingulum FA that correlated with hippocampal volume and verbal memory performance (Delano-Wood et al., 2012).

### 4.5 Obsessive compulsive disorder and depression

Both OCD and depression have been treated with cingulotomy (see Section 3.3) and both have been linked to reduced FA in frontal pathways, including the cingulum. An extensive meta-analysis of OCD from 22 data sets and 537 cases (Radua et al., 2014) found evidence of widespread white matter changes that were most robust for anterior midline tracts, including the cingulum. A separate review of 17 studies (Koch et al., 2014) concluded that reduced FA in the cingulum bundle and corpus callosum were the most frequent dMRI alterations in adult OCD patients. These diffusivity changes have been variously found in the right and left hemispheres (Table 2). Among these changes, the anterior cingulum can show reduced FA but increased white matter volume (Radua et al., 2014). Two particular issues are that these FA changes might be most evident when associated with medication (Radua et al., 2014; Fan et al., 2016) and that some OCD studies report increased FA and reduced diffusivity. For example, an increase in cingulum FA was reported in paediatric and adolescent cases (Koch et al., 2014), while Lochner et al. (2012) found that lower diffusivity in the right dorsal cingulum was associated with increased anxiety and depression in OCD (Table 2).

Bipolar depression, like OCD, is associated with widespread white matter changes, with decreased FA in frontal pathways (Heng et al., 2010), including the cingulum (Wang et al., 2008). These changes can lead to increased radial diffusivity and mean diffusivity in the right mid-dorsal part of the bundle (Benedetti et al., 2011). In a meta-analysis of 10 studies with 314 cases with bipolar disorder (Vederine et al., 2011) two clusters of reduced FA were found in the right hemisphere. One was close to the parahippocampal gyrus, the other close to the subgenual cingulate cortex. It seems likely that at least one of these clusters involves the cingulum. A second meta-analysis of dMRI findings in bipolar disorder (Nortje et al., 2013) implicated multiple frontal tracts, along with the corpus callosum. In that same analysis, three clusters of reduced FA were reported, one in the right posterior temporoparietal region and two in left cingulate regions (Nortje et al., 2013), the latter two sites presumably contributing to the cingulum bundle. In a further meta-analysis, Wise et al. (2016) considered FA in both depression and bipolar disorder. Both conditions showed reduced FA in the corpus callosum, while the reductions in FA in the left posterior cingulum were greater in bipolar than unipolar disorders (Wise et al., 2016).

A review of 35 DTI studies of major depressive disorder (Bracht et al., 2015) focussed on three reward pathways, including the cingulum. While changes to dorsal cingulum microstructure during acute depression were only reported in approximately half of the studies (Bracht et al., 2015), this may reflect the need to consider specific patient subgroups and the value of targeting particular pathways, e.g., frontal-amygdala connections within the cingulum (Cullen et al., 2010). Other issues concern the number of episodes of depression, age at onset, and duration of medication. For example, greater FA reductions were found in a number of tracts, including the dorsal cingulum (bilateral), in treatment resistant/chronic patients compared with first episode patients (de Diego-Adelino et al., 2014). Of particular relevance, therefore, is the finding of reduced cingulum FA in people at familial risk for depression (Bracht et al., 2015), with related studies indicating that reduced cingulum FA reflects a genetic vulnerability to depression (Huang et al., 2011; Keedwell et al., 2012).

### 4.6 Schizophrenia and autism spectrum disorders (ASD)

Both schizophrenia and ASDS are seen as developmental in origin with overlapping features. One overlapping feature concerns the status of the cingulum bundle, which often shows increased diffusivity and reduced FA in the anterior dorsal region of the tract (Table 2).

In schizophrenia, cingulum FA changes are part of more widespread disruptions to frontotemporal and frontolimbic pathways (Samartzis et al., 2014). Consequently, while cingulum abnormalities are most often seen near the genu, they may also occur in the parahippocampal cingulum (e.g. Whitford et al., 2014). Initially it appeared that the cingulum might be unaffected in first episode cases (Kyriakopoulos and Frangou, 2009), but Lee et al. (2013) reported reduced FA in both the left and right cingulum of 17 first episode patients. Similarly, bilateral increases in both axial and radial cingulum diffusivity were found in 18 first episode cases (Fitzsimmons et al., 2014), with radial diffusivity correlating with the severity of delusions of reference. Evidence that white matter, including the cingulum, is especially sensitive to the effects of aging in schizophrenia (Kochunov et al., 2013) may account for the greater prevalence of cingulum changes in those with well-established psychotic states. A further issue concerns the symptomatology in schizophrenia. For example, FA correlations were found between the right genu cingulum and hallucinations, while right parahippocampal cingulum status correlated with negative symptoms (Whitford et al., 2014).

To help separate cause from effect, there is much interest in those genetically ‘at risk’ of schizophrenia but with no overt psychotic symptoms. Only a minority of these familial studies find cingulum changes, with other structures more often affected (e.g., Clark et al., 2011; Hoptman et al., 2008; Karlsgodt et al., 2009; Maniega et al., 2008). A concern, however, is that these studies may be underpowered given the need to adjust for the degree of penetrance. More consistent cingulum bundle changes are seen in the 22q11.2 deletion syndrome (Kates et al., 2015), which carries a high risk of psychosis. While cingulum integrity was associated with positive prodromal symptoms of psychosis, these changes may be modified by medication (Kates et al., 2015). Carriers of variants of neuregulin 1 associated with schizophrenia may also show anterior cingulum dMRI changes (Wang et al., 2009). Overall, the cingulum is not uniquely affected in schizophrenia, but is part of a wider network of disrupted pathways. One priority is to determine how cingulum bundle changes contribute during prodromal states.

For ASD, relevant findings include a meta-analysis of 33 studies (Rane et al., 2015), as well as a review of 72 papers on white matter in this disorder (Ameis and Catani, 2015). Overall, there is a recurrent pattern of reduced FA and increased diffusivity, consistent with altered development (Ameis and Catani, 2015; Rane et al., 2015; Travers et al., 2012). Furthermore, as reduced frontal and posterior cingulate interconnectivity, as measured from resting state fMRI, is one the most reported effects (Rane et al., 2015), it should be no surprise that reduced FA in the dorsal cingulum is also a frequent feature of ASD (Rane et al., 2015; Shukla et al., 2011; Travers et al., 2012; but see Aoki et al., 2013; Table 2). In those ASD studies focussing on just the cingulum, reduced FA is again found (Ameis et al., 2013; Ikuta et al., 2014), with diffusivity increases (e.g., mean and radial) most evident in young (<11 years) ASD participants (Ameis et al., 2013).

Reflecting the involvement of the cingulum bundle in executive, social, and emotional processes, some studies have looked at correlations between cingulum FA and ASD symptoms. Such correlations were found for behavioural regulation scores (Ikuta et al., 2014), consistent with executive dysfunctions, though other studies have failed to find clear cingulum correlations (Rane et al., 2015). Arguably, more consistent cingulum FA correlations with executive function have been found in schizophrenia (Kubicki et al., 2009; Spalletta et al., 2014; Takei et al., 2009; see Table 2).

### 4.7 Attention deficit hyperactivity disorder

Structural MRI studies indicate that cingulate cortex volume is reduced in ADHD (Amico et al., 2011; Makris et al., 2007), while resting state disturbances suggest either hyperconnectivity or hypoconnectivity (Konrad and Eickhoff, 2010). Reviews of dMRI findings (Angriman et al., 2014; Konrad and Eickhoff, 2010) highlight the variable pattern of changes in which reduced FA in the cingulum is sometimes, but not consistently, reported (Makris et al., 2007; Pavuluri et al., 2009; Chiang et al., 2016; Table 2). The lack of reliable cingulum bundle changes may relate to the need to separate different subtypes of ADHD, as increased FA has been reported in the cingulum bundle in ‘combined’ but not ‘inattentive’ types of ADHD (Svatkova et al., 2016; Table 2).

Studies relating the severity of ADHD symptoms with dMRI indices (Angriman et al., 2014) reveal that clinical symptoms and executive dysfunctions correlate best with the status of fronto-striatal tracts, while cingulum bundle changes are less reliable. Nevertheless, Chiang et al. (2016) reported lower cingulum FA in ADHD that correlated with inattention, alongside the loss of cingulum correlations with executive functions seen in controls. In a study of 19 adolescents with ADHD that focussed specifically on the cingulum (Cooper et al., 2015), ADHD severity was associated with the status of the left cingulum close to the genu, but surprisingly, ADHD severity correlated with increased FA and reduced radial diffusivity (Cooper et al., 2015).

### 4.8 Post-Traumatic stress disorder

Dorsal cingulum changes occur in PTSD, as measured both by dMRI indices (Table 2) and tract volume (Daniels et al., 2013). There is evidence that tract volume changes are most frequent in the left hemisphere, while the dMRI changes occur in the dorsal cingulum (e.g., Sanjuan et al., 2013). In one study of PTSD survivors of a fire disaster, lower FA was found in the left rostral, subgenual, and dorsal cingulum (Kim et al., 2006). Following another disaster, survivors with PTSD had reduced FA in the right subgenual region (Zhang et al., 2011). Meanwhile clusters of dMRI changes were most evident in the cingulum and the superior longitudinal fasciculus in a meta-analysis of adult onset PTSD from seven studies (Daniels et al., 2013). While the largest cluster related to decreased FA in the right cingulum, different sections of the cingulum were associated with both increases and decreases of FA (Daniels et al., 2013; Table 2).

A potentially important factor concerns the nature of the trauma. A recent study of noncombat PTSD sufferers, found reduced FA in cingulate white matter just above the genu (Olson et al., 2017). In contrast, combat veterans with PTSD had increased cingulum FA (Davenport et al., 2015). Likewise, in studies of combat veterans, dorsal cingulum FA was positively associated with the persistence and severity of symptoms (Kennis et al., 2015, 2017). In the case of military personnel, there is the contributing complication that blast exposure (without PTSD) can affect cingulum FA (e.g., Ivanov et al., 2017; MacDonald et al., 2011), suggestive of axonal injury. These findings reinforce the value of separating different types of trauma associated with PTSD, while also showing that FA can increase as well as decrease in this condition.

### 4.9 Overview of psychiatric conditions and the cingulum bundle

Cingulum dMRI changes, which appear most consistently in OCD, ASD and PTSD, are concentrated in the dorsal cingulum (Table 2). A recurrent issue concerns the extent to which these dMRI changes reflect primary disorders of white matter, secondary white matter impairments following grey matter disorders, or both. This same issue of identifying primary dysfunction applies to the ‘default mode network’, an interlinked set of brain sites that often show reduced fMRI activity when performing cognitive tasks (Greicius, 2008, Greicius et al., 2009). The cingulum is centrally placed within this network, e.g., cingulum FA correlates with default-mode functional connectivity (van den Heuvel et al., 2008). Furthermore, alterations in the default mode network have been reported in schizophrenia (Öngür et al., 2010), ADHD (Konrad and Eickhoff, 2010), depression (Öngür et al., 2010; Zhu et al., 2012), PTSD (Bluhm et al., 2009), OCD (Peng et al., 2014), and ASD (Padmanabhan et al., 2017).

The pattern of overlapping white matter changes in different psychiatric conditions gives added interest to those studies that compare multiple disorders. A review of dMRI findings in bipolar disorders and schizophrenia (O’Donoghue et al., 2017), concluded that frontal disconnection is common to both illnesses. In bipolar disorders, abnormalities in interhemispheric and posterior limbic connectivity appeared more prominent, while schizophrenia was more linked with frontotemporal changes (O’Donoghue et al., 2017). Meanwhile, Nenadic et al. (2017) found evidence of increased radial diffusivity in the cingulum in schizophrenia but not bipolar disorder. Divergent changes in corpus callosum radial diffusivity pointed to further white matter differences in these two conditions (Nenadic et al., 2017). Finally, when ASD was compared with ADHD (Chiang et al., 2017), the ASD participants had reduced FA in six tracts, including the right cingulum bundle (parahippocampal). In the right parahippocampal cingulum and right arcuate fasciculus these FA values related to autistic social-deficit symptoms. Meanwhile, ADHD offers a different profile where raised FA can be found in some tracts (Davenport et al., 2010), and where more constrained cingulum diffusion can be positively correlated with the severity of symptoms (Cooper et al., 2015; but see also Chiang et al., 2016).

Studies of white matter status in psychiatric conditions are still in their infancy but dMRI data reveal overlapping patterns of fronto–cortical and fronto-limbic changes across a variety of disorders, with cingulum alterations a frequent component (Table 2). Together, these overlapping patterns support transdiagnostic views of psychiatric states. Further support comes from the finding that neonatal complications, a common risk factor for many neuropsychiatric conditions, can lead to cingulum bundle diffusion changes (Schiller et al., 2017a,b). Critical issues to be resolved include understanding the relationships between grey and white matter changes, the consequences of cingulum white matter changes on symptomatology, the significance of medication status, gender differences (Menzler et al., 2011), duration of the condition, and whether recognised sub-types within the various conditions have different profiles of white matter change. While most clinical studies that report a change in the cingulum, find increased diffusion associated with reduced FA, the opposite is sometimes found, e.g., in PTSD and ADHD. Understanding the underlying causes of these diffusions metrics is becoming increasingly important. For example, the finding that different parts of the cingulum may show opposite changes in diffusivity in both PTSD (Daniels et al., 2013) and OCD (Cannistraro et al., 2007) suggests that there can be opposing effects relating to which connections dominate the cingulum signal at that location.

## 5 Conclusions and future directions

This review began with a detailed analysis of the connections that comprise the rat and monkey cingulum bundle (Fig. 4, Fig. 5). The numerous similarities across species make it extremely likely that the human cingulum bundle contains the same, core connections as those found in other mammals. It is also presumed that the human cingulum bundle contains additional frontal and parietal connections (Fig. 1), e.g., as indicated by fMRI studies of effective connectivity. The review has also highlighted that of the many cingulum bundle fibres, it is principally the cortico-cortical connections and the subcortical efferents to the cingulate gyrus that join its long axis. Consequently, cingulum dMRI studies will often be biased to this group of connections. In contrast, cingulate projections to subcortical sites more typically cross the bundle and, hence, are less easy to detect.

A principal driver of the cingulum is the cingulate gyrus. It is, therefore, important to appreciate that the cingulate gyrus is associated with many functions. This point is emphasised in a meta-analysis of fMRI cingulate activations (Beckmann et al., 2009). In that study, area associations were found with at least seven different processes. These were: *Emotion*, dorsal anterior cingulate, as well as pregenual and subgenual cortex; *Reward*, dorsal anterior cingulate above the genu, as well as subgenual ventromedial frontal cortex; *Pain*, anterior and mid cingulate cortex (see also Vogt and Sikes, 2009); *Motor*, mid cingulate cortex (overlapping with ‘pain’); *Conflict*, anterior cingulate and paracingulate cortex, i.e., above the anterior and middle cingulum; *Error detection*, anterior cingulate and paracingulate cortex (overlapping with ‘conflict’); *Memory,* retrosplenial cortex, linked with hippocampal and parahippocampal connections (Beckmann et al., 2009). It is, of course, most likely that some processes, such as pain and emotion (Shackman et al., 2011), or motor control and aspects of attention (Paus, 2001) will overlap given their close relationships with one another.

This array of candidate functions, potentially supported by the cingulum, highlights the value of isolating particular pathways within the tract. At present, dMRI reconstructions fail to distinguish short association fibres, while also failing to identify the direction of a pathway. Furthermore, current cingulum subdivisions are based on the long axis of the bundle (Fig. 6). Although clearly better than treating the tract as a single entity, this approach still fails to isolate specific connections. At the same time, networks of interaction also need to be considered. One example concerns the ‘default-mode network’, which encompasses temporal, parietal, and frontal regions that are connected via the cingulum (Greicious et al., 2009). The growing appreciation that disturbances within this network contribute to many clinical states only reinforces the need to understand the cingulum. At the same time, while the default-mode network overlaps with the cingulum and many of its connections, there are additional connections within the cingulum that are presumably not part of this network, highlighting the challenge for dMRI research.

Table 3 brings together findings from multiple studies of cingulum bundle activity or dysregulation in order to highlight any recurring sets of functions. Those functions most strongly linked to different parts of the cingulum bundle are emotion (including social interactions), motivation, executive functions (including aspects of attention), pain, and memory. Consistent with how the core connections within the cingulum bundle are retained across species, it is notable that these functional categories have comparative support (Table 3). Furthermore, given the complex nature of the tract and its patterns of connectivity, it should be no surprise that these different attributes often interact with each other, blurring their distinctions.

**Table 3:** Major functions ascribed to various parts of the cingulum bundle. Column 2 indicates those cortical and subcortical connections most linked with the relevant function. Column 3 refers to that those subdivisions of the cingulum bundle (CB) particularly associated with that class of function. Column 4 gives examples of the relevant evidence from studies of rats (R), nonhuman primates (M), and humans (H). Note that at present there is a lack of evidence concerning selective cingulum bundle disruption in nonhuman primates.

Function	Principal connections	Suggested subsection	Evidence
Emotion (note link with pain as well as aspects of empathy)	Amygdala, medial and orbital prefrontal cortices, anterior cingulate cortex	Subgenual, anterior cingulate	R Anterior cingulate cortex lesions disrupt social responsiveness
			M Lesions involving CB cause subtle social deficits Anterior cingulotomy is partially effective in treating affective disorders
			Anterior cingulotomy is sometimes associated with decreased anxiety, depression, and hostility across clinical groups
			Affective disorders are associated with dMRI changes in white matter tracts, including the CB
			Emotion and reward related fMRI activity in subgenual and anterior cingulate cortex as well as amygdala.
Motivation	Anterior cingulate cortex, medial and anterior thalamus, medial and orbital frontal cortices	Anterior cingulate, subgenual	R Anterior cingulate lesions affect response cost judgements
			H Apathy is sometimes associated with anterior cingulotomy
			H Importance of orbital and medial frontal areas for hedonics
			H Reward related fMRI activations in ventromedial frontal and anterior cingulate areas
Executive function (including attention)	Dorsolateral and anterior cingulate cortices, medial and midline thalamus, ascending cholinergic fibres	Anterior cingulate, subgenual	Anterior cingulate lesions disrupt attentional tasks dependent on cholinergic inputs
			Rostral cingulate lesions involving the CB can disrupt some executive functions
			Anterior cingulotomy is associated with deficits in high level processing and selection
			dMRI correlations between anterior/dorsal cingulum and tests of cognitive control and executive function
			fMRI studies of cognitive control tasks
Pain	Midline and intralaminar thalamic nuclei, anterior cingulate cortex	Anterior cingulate, mid-cingulate	Blockade of CB leads to analgesia and delayed self-mutilation, whereas stimulation precipitates self-mutilation
			Anterior cingulotomy is partially effective in treating chronic pain
			Supracallosal cingulate fMRI activity in pain
Memory (including spatial processing)	Hippocampus, anterior thalamic nuclei, retrosplenial and parahippocampal cortices	Parahippocampal, retrosplenial	CB lesions can disrupt performance on spatial tasks involving allocentric cues
			Mild, inconsistent memory effects after supracallosal lesions that invade CB
			Anterior cingulotomy is associated with borderline deficits on some memory measures
			dMRI evidence of link between parahippocampal bundle and memory performance
			Memory loss and topographic amnesia is associated with retrosplenial cortex damage

To add to this complexity, there are other potential functions subserved by parts of the cingulum bundle. One such contribution relates to motor function, given the cluster of fMRI activations seen around the mid supracallosal cingulate cortex and adjacent motor areas (Beckmann et al., 2009). Indeed, the midcingulate area contains multiple motor areas (Vogt et al., 2005) that in monkeys are found in the lower bank of the cingulate sulcus (Morecraft and Tanji, 2009). A question remains, however, concerning the extent to which their connectivity involves the cingulum. While it is likely that their cortico-cortical and thalamo-cortical connections will often join or cross the bundle, additional research is required to specify the extent of cingulum involvement. Given the potential contributions of the cingulate motor areas to disorders such as OCD and ADHD, it would be valuable to have greater clarity on this issue.

The present review reveals a remarkable contrast: dMRI indices reveal an array of correlations with cognitive functions, which differ along the main axis of the pathway, yet lesion studies with nonhuman primates and clinical descriptions of cingulotomies typically fail to report robust changes in these same attributes. This mismatch is reinforced by the outcome of fMRI and PET studies, which repeatedly highlight the apparent importance of those cortical areas that contribute to the cingulum bundle for these same multiple functions (e.g., Beckmann et al., 2009; Cabeza and Nyberg, 2000; Shackman et al., 2011; Vogt, 2005). This mismatch presumably indicates that while cingulum fibres support these various abilities, the distributed nature of these functions, combined with the presence of alternate routes of communication, helps to ensure that the effects of cingulum disconnection often remain surprisingly mild and difficult to detect. Nevertheless, this same mismatch suggests that there is an important missing piece to the puzzle. It is, for example, clear from structural imaging that cingulotomies should create substantial cingulate disconnections (Heilbronner and Haber, 2014), yet the lack of resultant cognitive changes suggest otherwise. One option is to go back to patients with cingulotomies and use dMRI approaches to better understand the extent and nature of the disconnections they suffer post-surgery.

One example of this mismatch concerns learning and memory. Cingulum bundle lesions in humans at the level of the anterior cingulate cortex have little or no effect on standard tests of memory. A caveat concerns the need to know how cingulum bundle damage might affect the spontaneous recall of autobiographical memory, given how medial frontal damage can disrupt self-reference in memory recall (Kurczek et al., 2015) while cingulotomy can reduce spontaneous response generation (Cohen et al., 1999b). Nevertheless, the present null results are fascinating given the presumption that such cingulotomies will disconnect many anterior thalamic efferents (Heilbronner and Haber, 2014) and that the anterior thalamic nuclei are seen as critical components of the pathology responsible for diencephalic amnesia (Aggleton and Brown, 1999; Carlesimo et al., 2011). Such amnesias are very evident when assessed with standard memory tests, suggesting that the choice of test is not the only explanation.

There is an even stronger prediction that comparable tract damage at posterior cingulate (retrosplenial) levels will impair episodic memory. This outcome is to be expected as both retrosplenial damage and hippocampal damage can cause anterograde amnesia (Spiers et al., 2001; Valenstein et al., 1987; Vann et al., 2009), i.e., pathology in two regions linked by posterior parts of the bundle. Indeed, as cingulum bundle damage appears to be a consistent feature of retrosplenial amnesia (e.g., Yasuda et al., 1997; Heilman et al., 1990), its importance for episodic memory would seem highly likely. Meanwhile, unilateral retrosplenial damage, which presumably involves the cingulum, can cause topographic disorientation (Maguire, 2001; Takahashi et al., 1997), emphasising a specific role in landmark navigation (Auger et al., 2012).

While the evidence from posterior cingulotomy in humans is very limited, it does not indicate obvious memory loss (Turner, 1973). Furthermore, extensive lesions of the monkey anterior and posterior cingulate cortices, which involve the bundle, spare both spatial alternation and scene discrimination learning (Murray et al., 1989; Parker and Gaffan, 1997; but see Buckley and Mitchell, 2016), even though the latter task is regarded as a behavioural test of episodic memory (Gaffan, 1994). In contrast, both fornix and anterior thalamic lesions in monkeys impair these same behavioural tasks (Gaffan, 1994; Parker and Gaffan, 1997; see Table 1 for corresponding rat results). Furthermore, damage to the fornix, as well as the anterior thalamus, causes anterograde amnesia in humans (Aggleton and Brown, 1999; Aggleton et al., 2000; Carlesimo et al., 2011; Gaffan and Gaffan, 1991). Part of the explanation for the relative sparing after cingulum damage may come from evidence that retrosplenial amnesias can be transient (Kim et al., 2007; Saito et al., 2005), pointing again to surviving, complementary routes. Meanwhile, the apparent insensitivity of spatial memory tests for monkeys with cingulum damage suggests that behavioural tasks might need to be better tailored, e.g., to examine landmark usage.

The differences between the effects of fornix and cingulum bundle lesions are highly informative as both tracts contain many hippocampal and parahippocampal connections, yet only the fornix provides the direct hippocampal inputs to sites such as the anterior thalamic nuclei, the mammillary bodies, and the prefrontal cortex (Aggleton, 2012). The fornix also contains inputs to the hippocampus from sites such as the septum and supramammillary nucleus. The implication is that one or more of these fornix connections have a critical status with respect to episodic memory. At the same time, some of the most relevant cingulum connections, e.g., those involving prefrontal and parahippocampal areas, are supported by alternate routes, including some via the fornix. A prediction is that should both the fornix and the cingulum be damaged, the consequences for memory would be especially severe. A further implication is that fornix pathology might lead to a greater reliance on the retrosplenial and parahippocampal cingulum, as appears to occur in MCI (Metzler-Baddeley et al., 2012a; Ray et al., 2015). This apparent reliance is reflected in a greater use of ‘knowing’ rather than ‘remembering’ (Metzler-Baddeley et al., 2012a), consistent with a shift towards parahippocampal gyrus function (Aggleton and Brown, 2006). Unfortunately, the normal significance of the parahippocampal cingulum for memory remains largely unknown. While dMRI measures can correlate with memory scores (Sections 4.2,4.4), the position of the parahippocampal cingulum with respect to the adjacent angular bundle leaves concern about reconstructed fibre traces jumping pathways. This potential problem is exacerbated by the lack of conditions with selective pathology in the parahippocampal cingulum bundle.

There is a natural tendency to portray the cingulum as an inert pathway, i.e., its role is just to ensure the transmission of information. Our concept of white matter is, however, changing rapidly. It is now known that task learning can alter dMRI attributes (Hofstetter et al., 2013; McKenzie et al., 2014; Sampaio-Baptista et al., 2013; Xiao et al., 2016), suggesting a more dynamic role for white matter. It has, for example, been shown that two months of working memory training can cause dMRI changes in healthy young adults, including within the left parahippocampal cingulum (Caeyenberghs et al., 2016; Metzler-Baddeley et al., 2017). Amongst the many implications is the realisation that separating cause and effect when examining white matter status in clinical conditions is going to be highly challenging.

Finally, cingulum bundle changes occur across a remarkably wide range of clinical conditions. These changes relate to the structure, shape, and size of the tract, as well as to measures of microstructure derived from dMRI. One explanation for this array of dMRI effects stems from the wide range of connections within the cingulum. These same dMRI effects are rarely uniform along the length of the tract, presumably reflecting changing underlying connections. While many of the psychiatric conditions, as well as healthy aging, appear to be more related to changes in anterior parts of the bundle, neurodegenerative conditions such as Alzheimer’s disease appear linked with parahippocampal regions. One barrier to research in this area has been the inconsistent array of cingulum subdivisions used in dMRI studies and the associated inconsistent terminology. Based on the anatomy, we would advocate five cingulum subdivisions (Fig. 6D). Table 3 illustrates how these various cingulum subdivisions might contribute to different functions.

It is evident that our understanding of the human cingulum bundle will increasingly rely on dMRI studies. This reliance highlights the need to remember the shortcomings of this methodology (see Section 2.2.3). Within this approach, particular advances are likely to come from longitudinal studies designed to relate specific tract and connectivity changes to the genesis of disorders, while also considering the role of individual differences, such as genetic susceptibility and the impact of the environment, on cingulum bundle status. Within this framework, the remarkably lengthy maturation of the cingulum bundle poses additional challenges. The manner in which the bundle changes its dMRI properties throughout childhood, adolescence, and well beyond into middle age, also raises questions as to how these changes might relate to both normal and abnormal development. An example of the former would be the ways in which cingulum bundle changes contribute to the shifting landscape of adolescence (Blakemore and Choudhury, 2006), while remembering the complex issue of distinguishing cause from effect. Finally, a repeated message from this review is the need to place greater emphasis on the functions of the particular connections within the bundle. As Beevor (1891) appreciated long ago, the cingulum is not a unitary tract, and this may be the principal reason why the task of deconstructing its multiple pathways and related functions is still in its infancy.

## Funding sources

This work was supported by the Wellcome Trust (103722/Z14/Z) and the BBSRC (L021005/1) to JPA and by a joint Alzheimer’s Society and BRACE research fellowship to CM-B (grant 208/506818).

## Declarations of interest

None.
